# Considering Culture and Conflict: A Novel Approach to Active Bystander Intervention

**DOI:** 10.15766/mep_2374-8265.11338

**Published:** 2023-08-29

**Authors:** Kathryn C. Stephens, Tené Redman, Reneé Williams, Belinda Bandstra, Ripal Shah

**Affiliations:** 1 Fourth-Year Resident, Department of Psychiatry and Behavioral Sciences, Stanford Hospitals and Clinics; 2 Professor of Medicine, Department of Medicine, NYU Grossman School of Medicine; 3 Health Sciences Associate Clinical Professor, Department of Psychiatry and Behavioral Sciences, University of California, Davis; 4 Clinical Assistant Professor, Department of Psychiatry and Behavioral Sciences, Stanford University School of Medicine

**Keywords:** Microaggression, Discrimination, Culture, Upstander, Bias, Case-Based Learning, Psychiatry, Self-Assessment, Anti-racism, Diversity, Equity, Inclusion

## Abstract

**Introduction:**

Workplace microaggressions are prevalent in clinical settings and contribute to poorer mental health outcomes, as well as to higher rates of burnout for physicians and students experiencing them. While bystander workshops customarily provide guidance on direct interventions to a general audience, the literature does not yet address workshops in an academic setting that consider the individual's motivations and behavior patterns. We implemented a psychologically informed approach to microaggression training to increase participants’ understanding and willingness to undergo behavioral change.

**Methods:**

We created a survey that included 10 distinct scenarios of discrimination in the clinical setting. Participants’ willingness to intervene was assessed on a Likert scale prior to, then following, a 1-hour active bystander intervention workshop conducted virtually. The workshop outlined the role of culture and conflict management style in willingness to intervene. Four modes of intervention were outlined, including direct and indirect methods.

**Results:**

A total of 78 medical students, graduate students, residents, and faculty members participated in the workshop. Of those, we compared 68 individuals’ pre- and postworkshop responses to our questionnaire. We then focused on the 54 participants with no previous training in psychiatry or psychology. Utilizing a Wilcoxon signed rank test, we compared the average pre/post scores of willingness to intervene and found scores to have improved following workshop attendance (*Z* = −6.339, *p* < .001).

**Discussion:**

Our findings suggest that a psychiatrically informed and culturally sensitive approach to active bystander intervention workshops may promote upstanding more effectively in academic medicine.

## Educational Objectives

By the end of this session, learners will be able to:

1. Identify their own willingness to intervene when witnessing instances of discrimination in the clinical setting.
2. Consider cultural factors and personal conflict management style that may impact their willingness to be an active bystander.
3. Identify direct and indirect interventions for instances of workplace microaggressions.

## Introduction

Implicit biases are made explicit in a variety of ways. Discrimination has been conceptualized as occurring along a continuum from microaggressions to overt discrimination.^1^ Along this spectrum, microaggressions may stem from institutionalized and systemic teachings of bias or may be rooted in a personally mediated belief. A microaggression can be defined as a nuanced comment or action that expresses a prejudiced attitude toward a member of a marginalized group. Microaggressions may be intentional or unintentional and communicate a “hostile, derogatory, or negative attitude.”^2^ In contrast, overt discrimination involves explicit comments or behaviors against a group based on race, gender, sexual orientation, or other characteristics. Overt discrimination often occurs at a systems level, resulting in structural inequalities—including criminal justice and housing discrimination—that disadvantage members of minority groups.^3^

While more nuanced than overt discrimination, microaggressions have been associated with negative physical and mental health impacts for those who experience them.^4^ These include—but are not limited to—higher rates of anxiety, depression, and post-traumatic stress disorder, as well as hypertension and obesity.^5^ Medical providers and trainees who experience bias and discrimination at higher rates than the general population remain susceptible to these effects.^6^ A number of studies have noted the impacts of discrimination upon resident physicians, including higher rates of burnout and being made to feel other.^7,8^ Several moderators have been presented as a means of decreasing these adverse effects, including increased social support.^5^ Those who share their experiences of discrimination with others have lower rates of depression and lower blood pressure levels than those who do not vocalize their experiences.^9,10^

Bystanders who witness instances of discrimination and proactively respond are central to maintaining a workplace culture that pursues the well-being of all members. Yet studies have observed that the most natural bystander effect is that individuals become less likely to intervene as the number of people present at a situation increases, especially if the situation is nonurgent.^4,11^ To combat this tendency, microaggression training workshops have historically taught intervention techniques to assist bystanders to intervene more readily.^12,13^ Other *MedEdPORTAL* publications outline frameworks to conceptualize and respond to bias in the clinical setting.^3,14,15^ However, there are many intrapersonal dynamics and situational factors that influence an individual's willingness to step in when witnessing discrimination in the workplace. Psychologically informed models in diversity, equity, and inclusion (DEI) interventions that consider the individual's motivations and behavioral patterns are essential. Our team developed a workshop utilizing therapeutic psychological concepts, including the idea that it is necessary to understand one's own thoughts and motivations in order to create lasting behavioral change.

By teaching a framework for understanding cultural concepts, conflict management styles, and distinct intervention methods, our workshop aims to minimize the bystander effect by equipping participants across the spectrum of training and medical practice—from medical students to faculty. To understand their own motivation or hindrance to becoming an active bystander, participants are tasked with self-reflection exercises and receive practical tools for doing so by utilizing both direct and indirect methods of intervention. We believe that psychologically informed approaches to microaggression training can increase participants’ understanding and willingness to undergo behavioral change.

## Methods

This instructor-led educational workshop was developed as an initiative of the Race and Mental Health Lab in the Stanford University School of Medicine's Department of Psychiatry and Behavioral Sciences. The workshop included direct instruction with PowerPoint slides (Appendix A), self-reflection questions presented in a Zoom poll, and case discussions as a large group. We partnered with the Stanford Medicine Office of Faculty Development and Diversity to learn from its experience in both developing and facilitating DEI workshops. We developed a survey that featured 10 distinct scenarios of discrimination, including microaggressions and overt discrimination, in the clinical setting and assessed the participant's willingness to intervene both prior to and following our workshop. Because the workshop was conducted during the COVID-19 pandemic, it was offered virtually via Zoom. Five 1-hour sessions were held over 2 weeks. Participants were given a $20 Amazon gift card for their involvement.

An invitation to attend the workshop was sent via email to all members of the Department of Graduate Medical Education and to all students in the School of Medicine at Stanford University. A total of 78 medical students, residents, fellows, and faculty attended one of the five 1-hour workshops.

Of these participants, 99% completed the preworkshop survey, and 92% completed the postworkshop survey. Fifty-four percent identified as a resident or fellow, 28% identified as a medical student, 13% identified as faculty, and 4% identified as a graduate student. Approximately 67% identified as belonging to a minority group, and 22% indicated some level of training in psychiatry or psychology. Of those who participated, we were able to compare 68 individuals’ pre- and postworkshop responses to our upstander questionnaire. We focused on the 54 participants who had no previous training in psychiatry or psychology to assess their response to our workshop. Inclusion criteria were any medical student, graduate student, resident physician, fellow physician, or faculty member at the Stanford University School of Medicine who received a participant registration email via the Department of Graduate Medical Education or the Stanford University School of Medicine. Participants with a self-reported background in psychiatry or psychology were excluded from the data analysis, although they were able to participate in the workshop and surveys (Table 1).

**Table 1.**
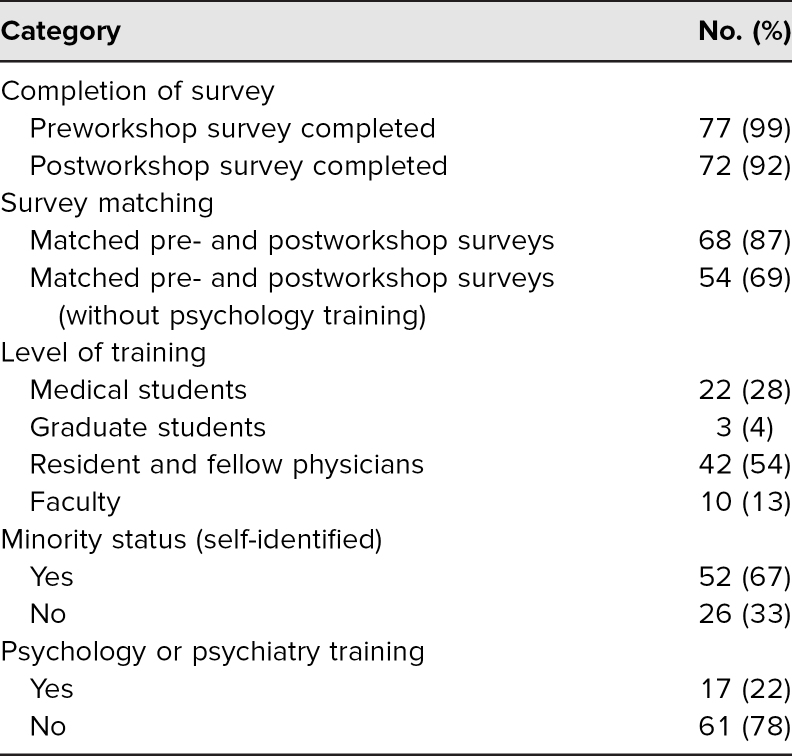
Demographics of Workshop Participants (*N* = 78)

### Workshop Timeline

• 5 minutes: preworkshop survey.
• 10 minutes: impact of microaggressions in the workplace.
• 15 minutes: explanation of cultural concepts and conflict management styles.
• 10 minutes: teaching active bystander intervention methods.
• 10 minutes: scenario examples and large-group discussion.
• 5 minutes: postworkshop survey.
• 5 minutes: optional group discussion/workshop feedback.

At the beginning of the workshop, participants were given 5 minutes to take the preworkshop survey (Appendix B). Developed by a focus group of psychiatry residents and faculty at our university-based psychiatry training program, the survey featured a diverse representation of microaggressions in the clinical setting, including racial, religious, gender-based, and other forms of discrimination representative of real-life scenarios. The preworkshop survey asked participants to indicate their level of training and whether they identified as belonging to a minoritized group as a means of evaluating the effectiveness of the workshop among different groups. We also inquired whether participants had previous training in psychiatry or psychology, as they may have had a greater degree of familiarity with the psychological concepts implemented in our training, which could skew the degree of impact we might otherwise observe.

After participants had completed the preworkshop survey, we provided approximately 20 minutes of teaching from the PowerPoint slides (Appendix A). We first taught participants about the detrimental impacts of microaggressions in the workplace. We then normalized feelings of uncertainty about intervening in the moment, citing power dynamics, feeling unprepared, and fears of repercussion. We presented Hofstede's cultural concepts^16^ and a framework of conflict management styles adapted from the Thomas-Kilmann Conflict Mode Instrument.^17^

The cultural concepts^16^ included the following:

• Power distance: the degree to which less powerful individuals accept and expect an unequal distribution of power.
• Individualism versus collectivism: the degree to which individuals act in the service of their own interests compared to those of the community.
• Task orientation versus person orientation: the degree to which individuals are motivated by success and achievement compared to group harmony and caring for the weak.
• Uncertainty avoidance: the degree of risk aversion an individual may feel given their cultural background.

The conflict management styles^17^ included the following:

• Directive: comfortable addressing issues head-on, even in times of conflict.
• Avoidant: using indirect methods (e.g., humor) to solve conflict or diffuse a situation.
• Harmonizing: collaborating and accommodating to reach a compromise for the good of the group.

After each cultural concept and conflict management style had been shared, participants were invited to participate in a live Zoom poll to indicate how they identified with each of the four cultural concepts (e.g., being someone with a high or low power distance), as well as their preferred conflict management style (Appendix C). We next taught the 4Ds model of active bystander intervention: distract, direct, delegate, and delay.^18^

At the conclusion of the workshop, two of the 10 scenarios from the questionnaire were read aloud. Participants responded either in the Chat box or aloud with their initial impressions of the scenario, discussing phrases that they might say in the moment to intervene. The discussion highlighted both direct and indirect methods of intervention, both in the moment and after an incident. Following the workshop, participants completed a postworkshop survey (Appendix D), answering questions regarding their willingness to intervene for the same scenarios as those presented in the preworkshop survey. Feedback regarding the workshop was also provided in the postworkshop questionnaire. This project was determined to be exempt from review by the Stanford University Institutional Review Board.

## Results

For each of the 10 scenarios in the survey, participants were able to select willingness to intervene from a 5-point Likert scale. Note that a lower score corresponds to higher willingness to intervene, so that a decrease in score corresponds to an increased willingness to intervene. Aggregate data were analyzed and then grouped by level of training (medical student, graduate student, resident/fellow, or faculty) to determine if this had an impact on participants’ willingness to intervene. Of the 54 participants with matched surveys and no prior training in psychology/psychiatry, 19 were medical students (35%), two were graduate students (4%), 24 were residents and fellows (44%), and eight were faculty (15%). One participant did not include level of training (2%). Descriptive statistics were used to analyze the mean and distribution of the dataset. Pre- and postintervention means were compared using the Wilcoxon signed rank test. A nonparametric test was selected as the data did not follow a normal distribution.

When analyzing the 54 participant responses, individual participants’ average willingness to intervene for all 10 scenarios ranged from 1.0 to 3.3 in the preworkshop survey and from 1.0 to 2.4 in the postworkshop survey. The overall mean of the preworkshop survey was 2.0 (*SD* = 0.6), which corresponded to *likely to intervene.* The overall mean willingness to intervene decreased to 1.3 (*SD* = 0.3) in the postworkshop survey, which was closer to being reflective of *extremely likely to intervene.* The average mean willingness to intervene was compared between the pre- and postsurvey workshops and found to be statistically significant (*Z* = −6.339, *p* < .001). This initial finding suggests that utilizing a psychiatrically informed and culturally sensitive training lens that provides a multitude of upstander intervention approaches may be an effective training method to enhance active bystander intervention.

When considering participants’ level of training, medical students (*N* = 19) had an initial average willingness to intervene of 2.2 (*SD* = 0.6) and a posttraining average willingness of 1.4 (*SD* = 0.4). Residents and fellows (*N* = 24) had an initial average willingness to intervene of 2.0 (*SD* = 0.6) and a posttraining average willingness of 1.3 (*SD* = 0.3). Faculty (*N* = 8) had the lowest initial score for willingness to intervene (corresponding to being most likely to intervene), with an average of 1.7 (*SD* = 0.5), and a posttraining average of 1.3 (*SD* = 0.3). Reported willingness to intervene increased amongst all analyzed groups in the postworkshop survey (Table 2). Using a Wilcoxon signed rank test, we found the increase in reported willingness to intervene to be statistically significant in all groups (medical students, *p* < .001; residents and fellows, *p* < .001; faculty, *p* = 0.03), with the exception of the graduate student cohort, which was not analyzed due to only having two members in the group.

**Table 2.**
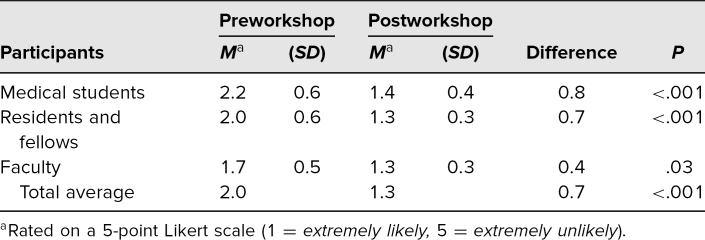
Mean Participant Scores for Willingness to Intervene on Pre- and Postworkshop Surveys^a^

Medical students were the least willing to intervene, yet they also were the group whose mean score demonstrated the greatest change toward willingness when comparing the pre- and postworkshop surveys (Table 2). Faculty, conversely, were more willing to intervene than other groups and had the smallest degree of change when comparing pre- and postworkshop surveys (Table 2).

## Discussion

Microaggressions in the workplace are highly prevalent and may contribute to poorer mental health outcomes, as well as higher rates of physician burnout for those who experience them.^5^ When acts of discrimination are not addressed, they can also negatively impact the culture within the work environment. Given these potentially devastating impacts on individuals and work communities, we considered the barriers to intervening and recruited principles of psychology to motivate greater willingness to address discrimination in the workplace.

While a multitude of bystander engagement workshops have been developed in recent years, our curriculum was devised in response to frustration surrounding existing trainings that often utilize prescriptive instruction and due to a desire for more individualized intervention methods. With insight-oriented learning of the material, we believed that participants would likely be better able to personally relate to the information learned, as it would create a greater sense of emotional salience with the proposed scenarios. Engagement in the workshop surpasses purely cognitive reframing and attempts to touch on emotional and personal connections to this topic. While general bystander trainings and our self-reflection-based bystander training can both be beneficial, our workshop aims to expand the application of psychological factors used to expedite behavioral change on a systemic level, much as is done with psychotherapy patients on an individualized level by trained clinicians.

The workshop aims to provide skills to those who feel empowered to intervene, while also offering a multitude of intervention strategies to select from for those who may not yet feel empowered. Our positive findings, notwithstanding the addressable concerns below, highlight the idea that despite limited generalizability of our results to other institutions or varying training levels, this workshop appears to be effective for our participant population.

Across all groups of training, self-reported willingness to intervene significantly increased upon completion of the workshop. We believe this effect was exhibited because our curriculum uniquely incorporates a principal idea in psychology: that change is motivated by insight into one's own behavioral patterns and cultural influences. Participants reflect preemptively on which modality is suited to their value system, guided by cultural factors and their preferred conflict management style. Our results suggest that by exposure to this framework, participants may be more willing to apply a chosen intervention method. The workshop also presents several direct, in-the-moment strategies alongside indirect methods of intervening. The intention here is to provide several intervention options to consider, to minimize feeling overwhelmed when witnessing a microaggression. Several participants noted in feedback that knowing how to respond in the moment is a struggle faced by an active, rather than passive, bystander.

Because our surveys rely on participants’ self-assessment rather than clinically observed interventions, self-reported increased willingness to intervene must be interpreted with caution. This workshop uses hypothetical case examples, which leaves the question of whether participants, when faced with such situations in real life, will apply what they have learned. Sending a follow-up questionnaire to participants at a designated time after workshop completion (e.g., 6 months) may be beneficial in determining the impact of the workshop on actual bystander interventions (rather than purely self-reported willingness to intervene). Moreover, determining the impact of psychologically informed training methodologies could be undertaken by recruiting a control group of participants randomized to a more prescriptive instruction format for bystander training that does not discuss topics of emotional salience (e.g., individuals’ cultural background or personal preferences).

Numerous participants appreciated the succinct 1-hour length of the workshop. The timing of workshops was conducive to medical students, residents, and faculty, who attended at convenient times such as after work or during a lunch break, with varying days of the week offered to best account for inpatient, outpatient, and operating room schedule differences amongst providers and specialties.

Upon completion of the first workshop, several participants requested additional time to discuss unique scenarios. Given this feedback, adjustments in the schedule were made so that subsequent sessions included three case discussions following the presentation. Because the data were not compartmentalized by date of workshop, we were unable to determine if these added case discussions positively impacted reported willingness to intervene on the postworkshop survey; however, we suspect that additional case explanations and more time for brainstorming communication tactics in a large-group format were likely useful in allowing participants to feel prepared with a diverse array of intervention techniques. Given the sensitive nature of the material discussed, we chose to avoid small-group role-play as a teaching modality, opting instead for large-group discussion. Our intention was for role-play to occur internally in the privacy of participants’ minds as they imagined their responses in real life to the scenarios displayed.

Our finding that medical students were initially more reticent to intervene than faculty members may highlight the role of power dynamics in an individual's willingness to intervene. As previously conceptualized by Ehie, Muse, Hill, and Bastien, it may be the case that the leadership skills required to be an active bystander are developed over time, like “any other clinical skill development.”^4^ Thus, our results may demonstrate a ceiling effect with faculty already inclined to intervene prior to this event. Additionally, because medical students had the most demonstrative shift from pre- to postworkshop mean scores, there may be compelling reasons to incorporate training on power distance and bystander interventions earlier in trainees’ careers.

Because recruitment of workshop participants was undertaken during the COVID-19 pandemic, in-person recruitment tactics (e.g., flyer postings and in-person announcements) were inaccessible. We instead recruited participants through invitation on community email lists, including broader student and trainee forums, and also diversity-focused groups. Our emails may have faced possible low readership given the large number of emails sent, as well as higher likelihood of being opened and reviewed by those who are already interested in DEI-related work. Recruitment by email may have limited our ability to effectively enroll additional interested participants, and recruitment from diversity-focused groups may have biased our findings by including those already motivated to make behavioral changes in this topic area.

As specific invitations were not directed towards any faculty email lists, those faculty who attended the workshop likely did so after hearing about it from trainees, which created an ascertainment bias. The voluntary nature of the workshop, albeit incentivized by an Amazon gift card, introduced volunteer bias as this factor may have increased the likelihood that participants with more commitment to or knowledge of this topic attended. Not all participants completed the preworkshop survey, while several others did not complete the postworkshop survey, and a few did not enter the same six-digit alphanumeric code in their pre- and postworkshop surveys, creating challenges to match the data and contributing further selection bias in the end dataset. Minimizing these biases could be achieved by broadening the reach for participant recruitment, submitting the workshop to be included in required department-wide meetings or residency didactic sessions, repeating instructions to participants for the surveys, and matching pre- and postworkshop surveys in real time. A notable concern of many diversity-focused workshops, including ours, is that those most interested are often repeat attenders; accessing those who have limited background in this area can prove difficult. Therefore, this workshop being required would assist in ensuring that the information is also received by those who may be less aware of the impacts of discrimination or are not actively pursuing anti-racism workshops.

An intentional aspect of the project design was that in both the pre- and postworkshop surveys, we deliberately did not specify or define what it meant to intervene in a scenario of discrimination. Rather, participants needed to rely on their own definition of intervention beforehand; afterwards, they were able to consider the methods taught in the training. Given this freedom of interpretation, we were not able to collect clear data on how participants would in fact intervene (i.e., directly or indirectly). The training emphasized the value of indirect interventions throughout the teachings but also highlighted the importance of speaking up in the moment as a key component of creating an inclusive workplace culture. Therefore, it is possible that direct methods had greater gains in intervention adoption than indirect methods.

The decision to withhold questions about specific demographic data was made to protect the privacy of the participants. Inquiring about a participant's minority identity as well as their area of specialty could unintentionally compromise their identity, particularly in a small training program. However, this decision limited our ability to perform an extended analysis of our results, as we had only self-reported minority status as a binary data point (yes or no), without further delineation into specific ethnicity groups or LGBT status, for example.

While participants provided feedback that they appreciated the 60-minute session length, extending the workshop to 90 minutes could allow for some of the issues in design listed above to be adequately addressed. Participants also provided feedback suggesting additional time to practice the intervention methods taught, as well as time to discuss and apply them to a larger selection of scenarios than the two that were highlighted. A 90-minute workshop would also allow opportunities for small-group discussions and to discuss communication techniques for all 10 of the survey scenarios.

Following the initial workshop series, we received requests from multiple clinical departments at the Stanford University School of Medicine for this curriculum to be presented to large groups as a required session for faculty, staff, and/or trainees. This provided an opportunity to reach participants with varying levels of awareness and understanding of microaggressions, as stated above. It also allowed for specialized adaptations to the curriculum, applicable to the department or specialty area being instructed. Presentations were slightly edited to include case examples that were most relevant to their area of practice. Faculty-specific presentations were also able to touch upon the unique roles and responsibilities of medical students, residents, fellows, health care staff, and faculty in an effort to address the inherent power distance perceptions outlined above.

Our workshop aims to expand participants’ concept of what counts as intervention. In doing so, we train participants to intervene more readily by promoting a greater self-awareness of their cultural influences and conflict management styles and by teaching both direct and indirect means of intervention. Psychologically informed methods and self-reflective exercises can be more widely utilized in DEI-focused workshops, such as bystander interventions for microaggressions. Active bystander trainings are designed to encourage mental flexibility and promote behavioral change in their participants; psychiatrists and psychologists are experts in doing this work on a daily basis with individuals. Continuing to integrate the fields of psychology and DEI work, as initiated here, may be powerfully synergistic in promoting true individual behavioral change on a large scale and on a systems level.

## Appendices


Upstander Bias Workshop.pptxUpstander Preworkshop Survey.docxZoom Poll Questions.docxUpstander Postworkshop Survey.docx
*All appendices are peer reviewed as integral parts of the Original Publication.*
