# Supplementary material for: Considering Culture and Conflict: A Novel Approach to Active Bystander Intervention
Source: MedEdPORTAL. 2023 Aug 29;19:11338. doi: 10.15766/mep_2374-8265.11338 (PMC10462770; doi:10.15766/mep_2374-8265.11338)
Supplement: Supplementary file 1 — Upstander Bias Workshop.pptxUpstander Preworkshop Survey.docxZoom Poll Questions.docxUpstander Postworkshop Survey.docx [file mep_2374-8265.11338-s001.zip › C. Zoom Poll Questions.docx]

Poll questions:

What do you identify as?

- High power distance
- Low power distance
- Somewhere in between

What do you identify as?

- Individualistic
- Collectivistic
- Somewhere in between

What do you identify as?

- Task Oriented
- Person Oriented
- Somewhere in between

What do you identify as?

- High risk avoidance
- Low risk avoidance
- Somewhere in between

What is your approach to conflict?

- Avoidant
- Harmonizing
- Directive
