# Supplementary material for: Considering Culture and Conflict: A Novel Approach to Active Bystander Intervention
Source: MedEdPORTAL. 2023 Aug 29;19:11338. doi: 10.15766/mep_2374-8265.11338 (PMC10462770; doi:10.15766/mep_2374-8265.11338)
Supplement: Supplementary file 1 — Upstander Bias Workshop.pptxUpstander Preworkshop Survey.docxZoom Poll Questions.docxUpstander Postworkshop Survey.docx [file mep_2374-8265.11338-s001.zip › A. Upstander Bias Workshop.pptx]

## Slide 1
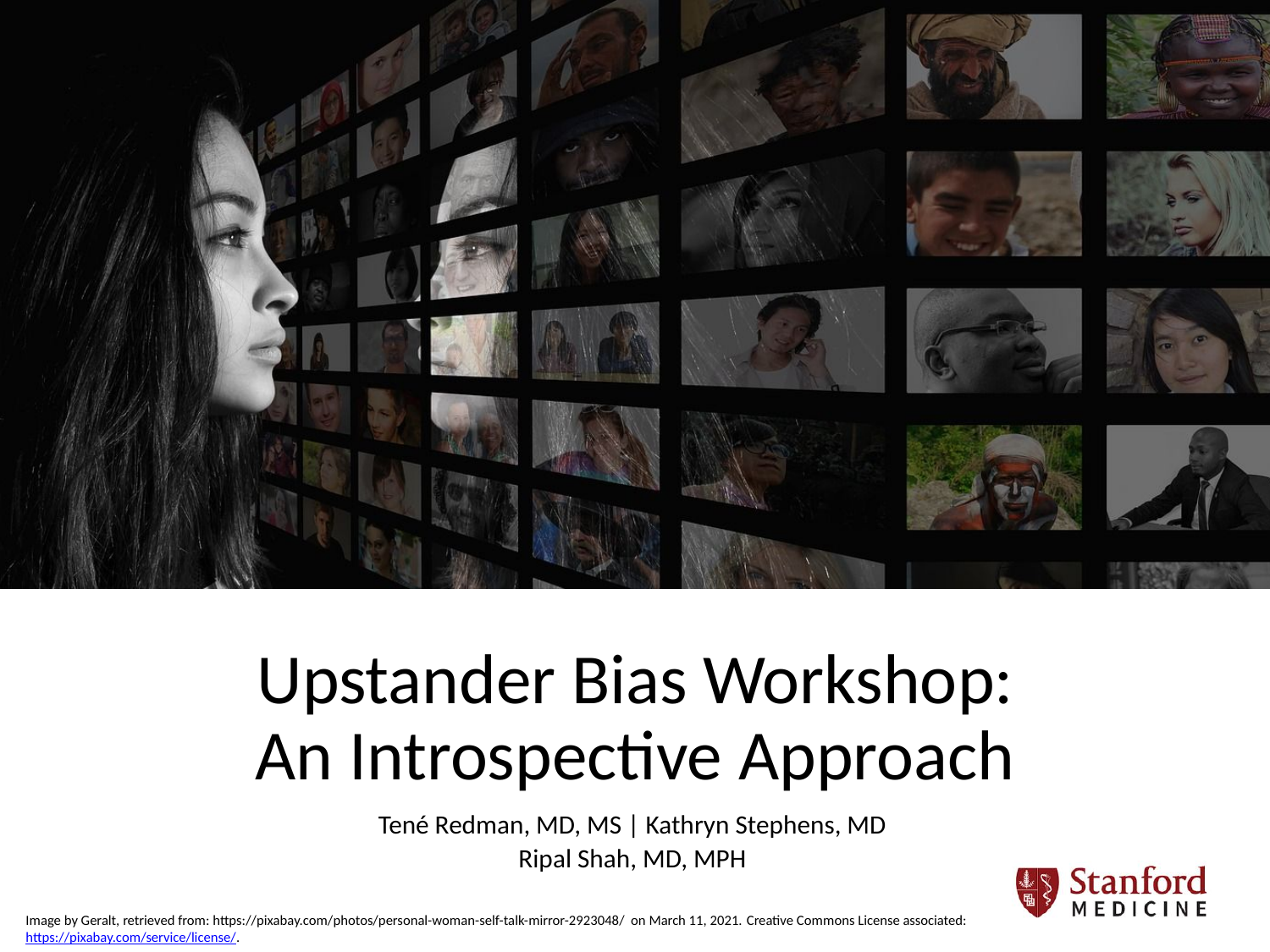

# Upstander Bias Workshop: An Introspective Approach
Tené Redman, MD, MS | Kathryn Stephens, MD
Ripal Shah, MD, MPH
Image by Geralt, retrieved from: https://pixabay.com/photos/personal-woman-self-talk-mirror-2923048/ on March 11, 2021. Creative Commons License associated: https://pixabay.com/service/license/.

## Slide 2
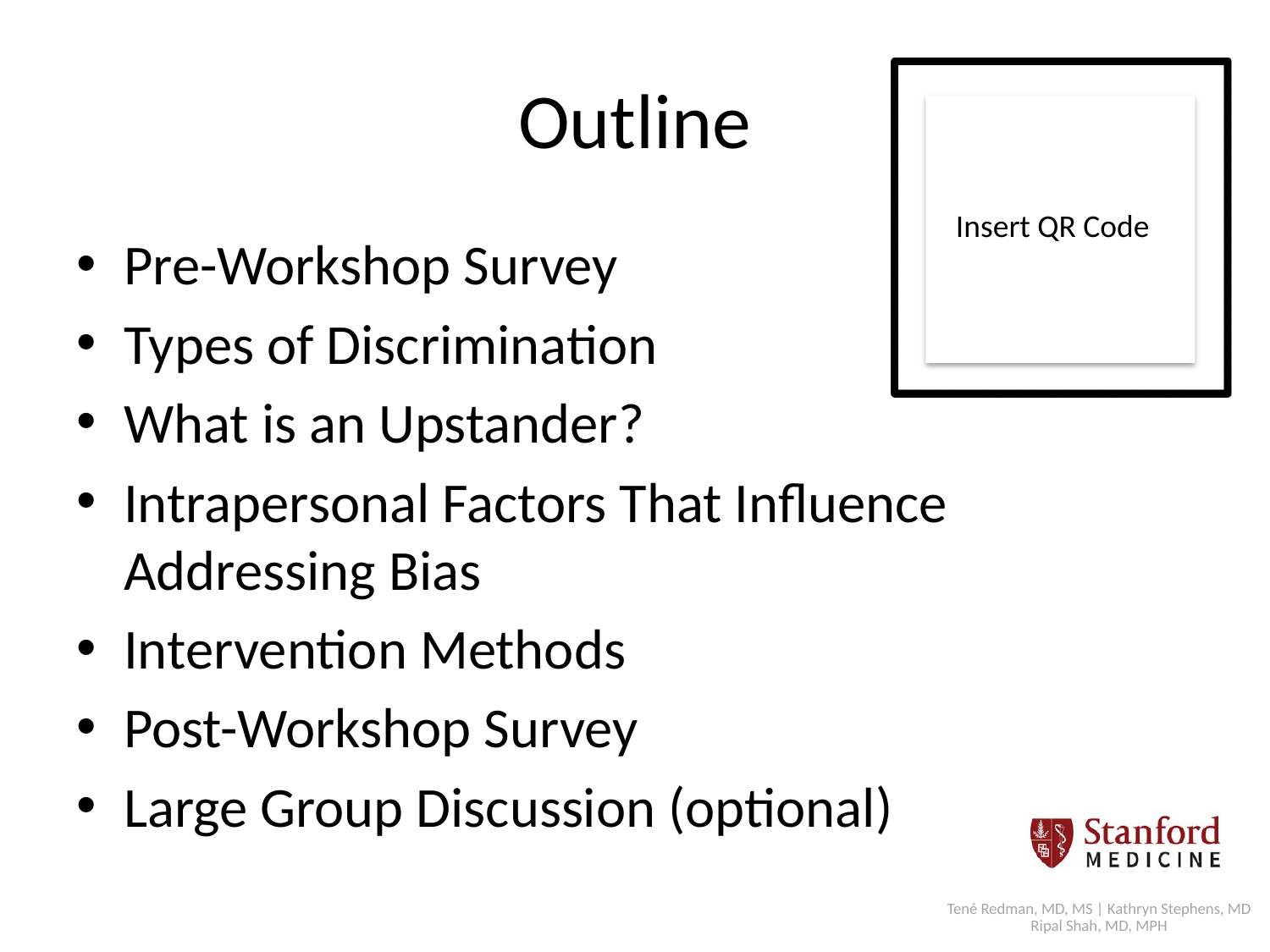

# Outline
Insert QR Code
Pre-Workshop Survey
Types of Discrimination
What is an Upstander?
Intrapersonal Factors That Influence Addressing Bias
Intervention Methods
Post-Workshop Survey
Large Group Discussion (optional)
Tené Redman, MD, MS | Kathryn Stephens, MD
Ripal Shah, MD, MPH

## Slide 3
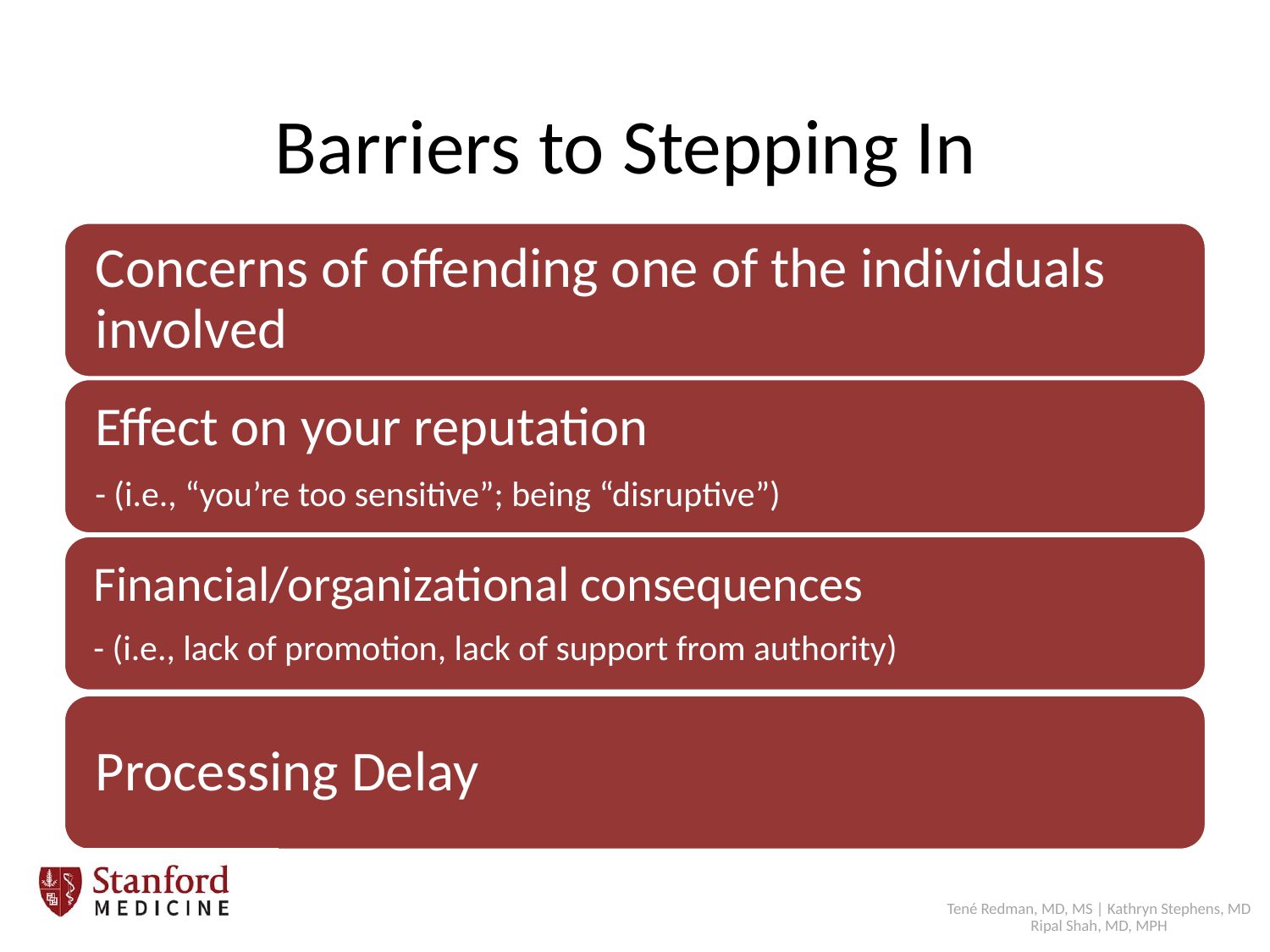

# Barriers to Stepping In
Tené Redman, MD, MS | Kathryn Stephens, MD
Ripal Shah, MD, MPH

## Slide 4
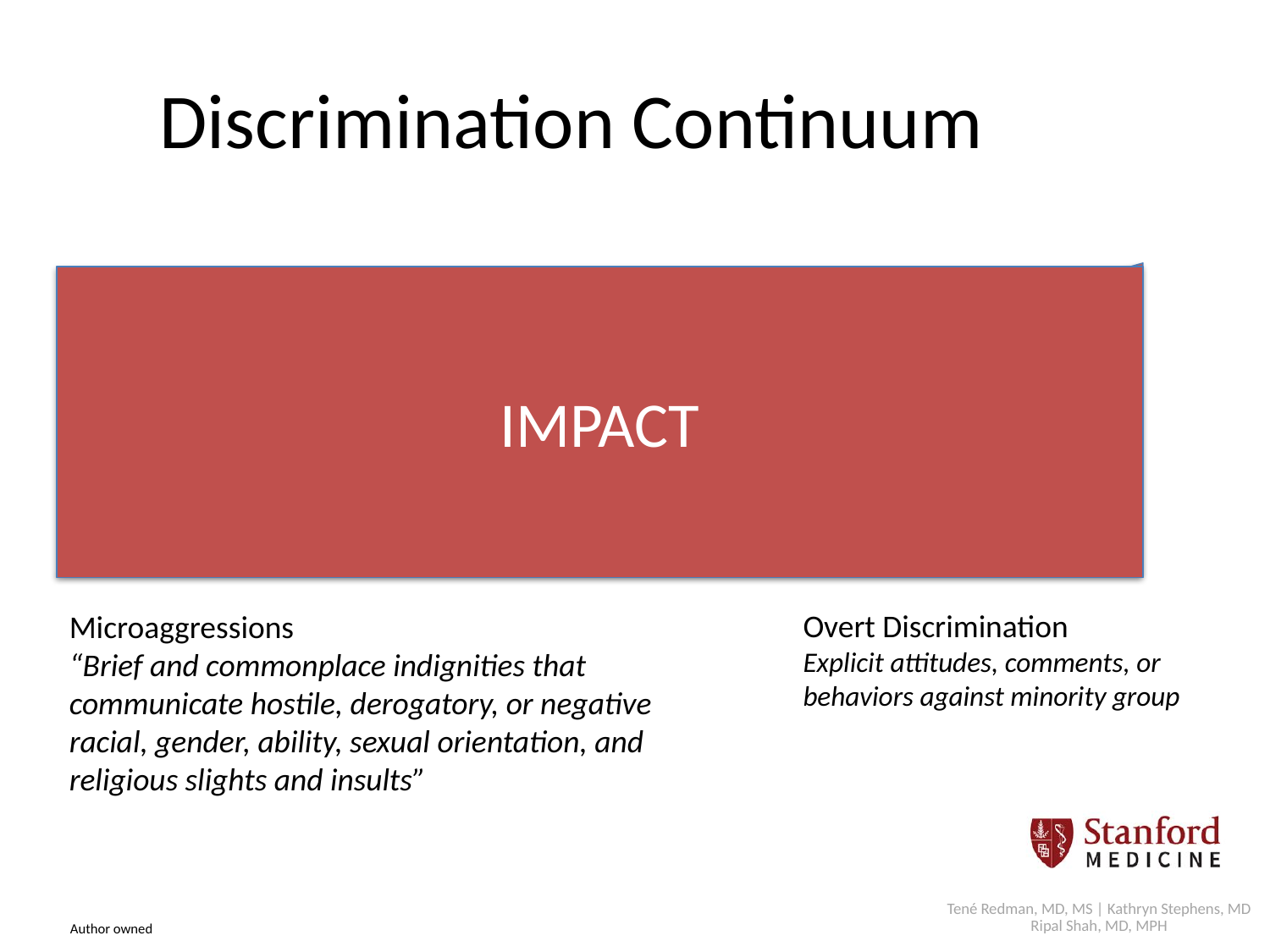

Discrimination Continuum
IMPACT
visibility
Overt Discrimination
Explicit attitudes, comments, or behaviors against minority group
Microaggressions
“Brief and commonplace indignities that communicate hostile, derogatory, or negative racial, gender, ability, sexual orientation, and religious slights and insults”
Tené Redman, MD, MS | Kathryn Stephens, MD
Ripal Shah, MD, MPH
Author owned

## Slide 5
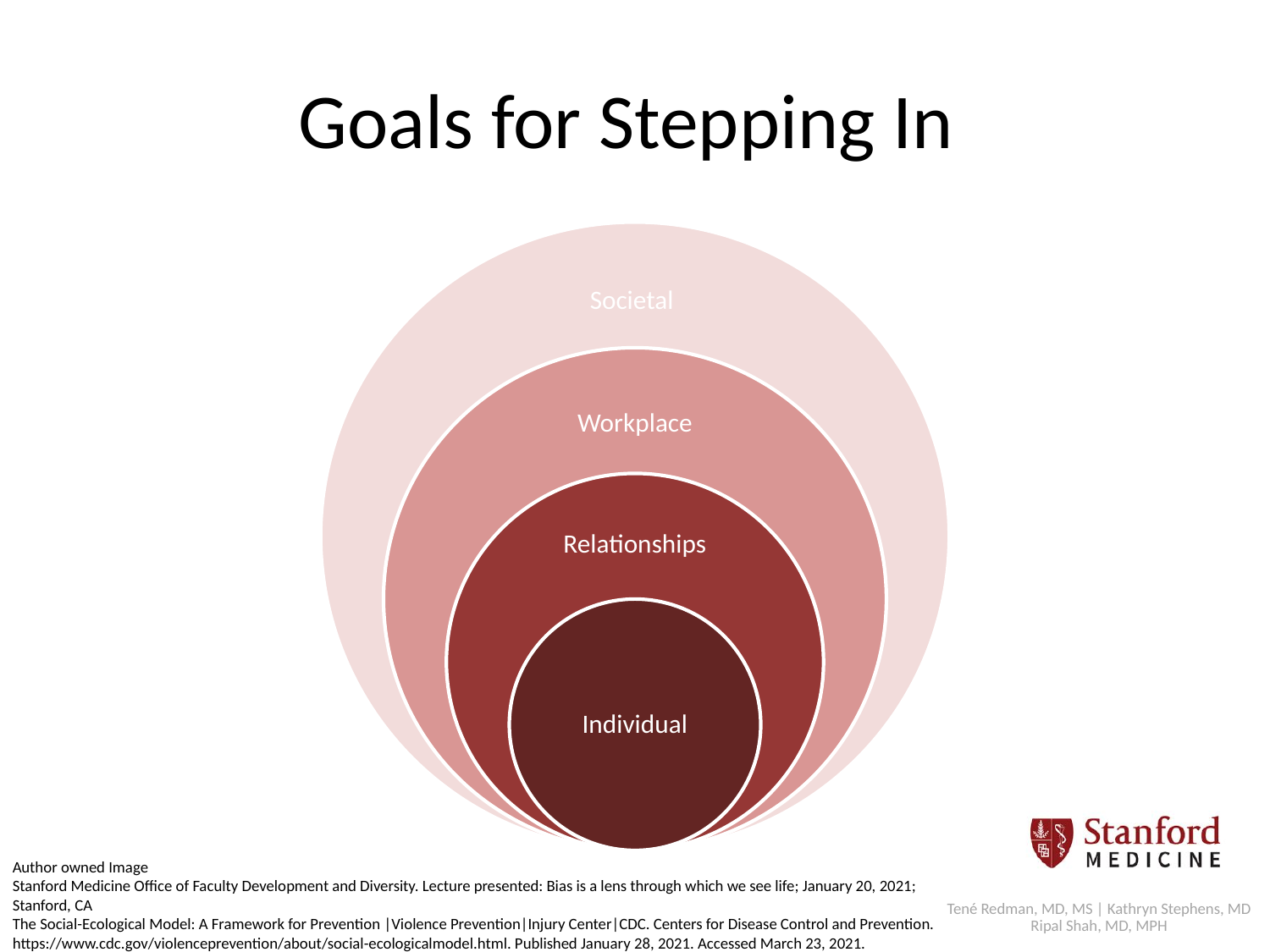

# Goals for Stepping In
Author owned Image
Stanford Medicine Office of Faculty Development and Diversity. Lecture presented: Bias is a lens through which we see life; January 20, 2021; Stanford, CA
The Social-Ecological Model: A Framework for Prevention |Violence Prevention|Injury Center|CDC. Centers for Disease Control and Prevention. https://www.cdc.gov/violenceprevention/about/social-ecologicalmodel.html. Published January 28, 2021. Accessed March 23, 2021.
Tené Redman, MD, MS | Kathryn Stephens, MD
Ripal Shah, MD, MPH

## Slide 6
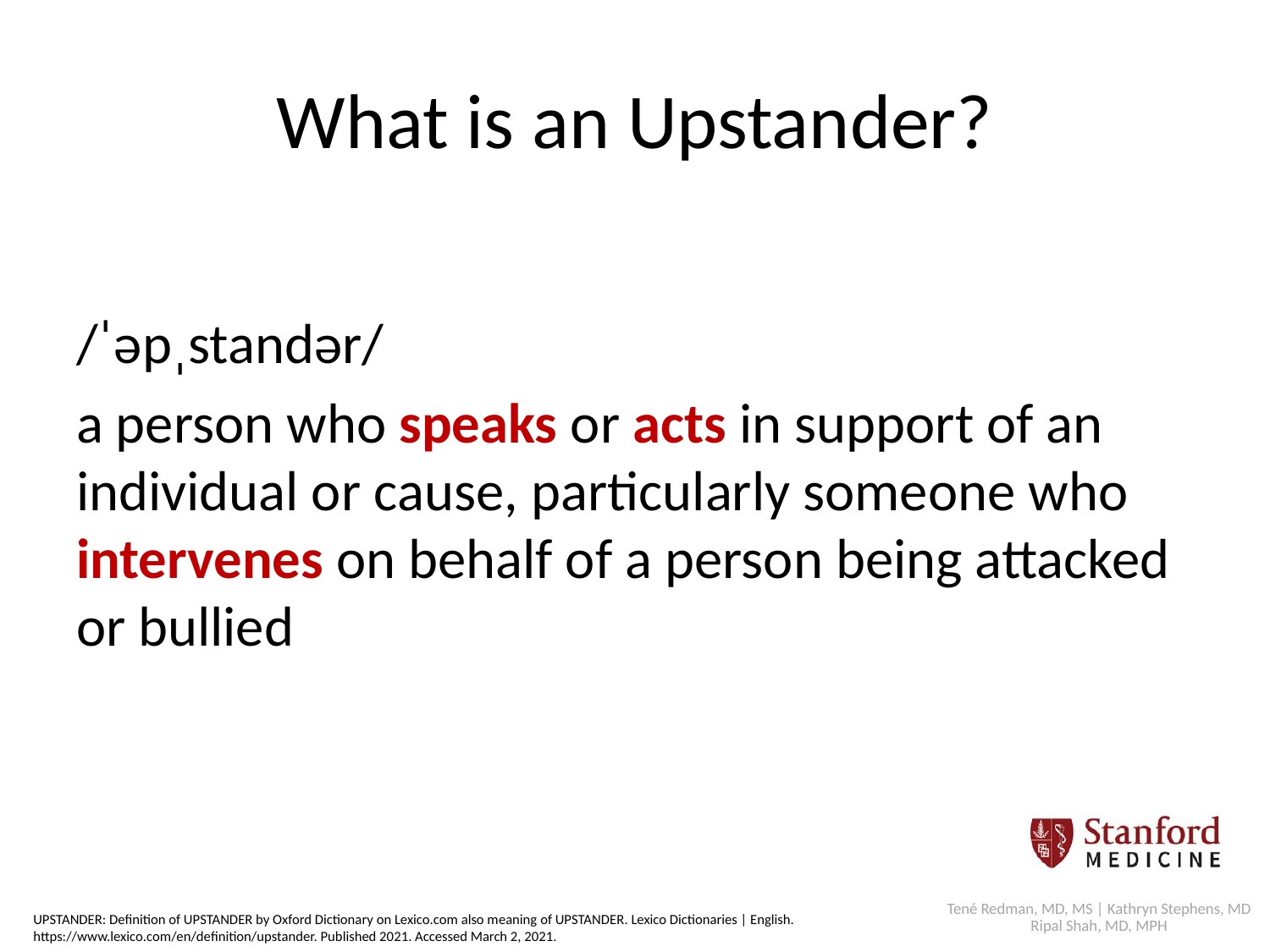

# What is an Upstander?
/ˈəpˌstandər/
a person who speaks or acts in support of an individual or cause, particularly someone who intervenes on behalf of a person being attacked or bullied
Tené Redman, MD, MS | Kathryn Stephens, MD
Ripal Shah, MD, MPH
UPSTANDER: Definition of UPSTANDER by Oxford Dictionary on Lexico.com also meaning of UPSTANDER. Lexico Dictionaries | English. https://www.lexico.com/en/definition/upstander. Published 2021. Accessed March 2, 2021.

## Slide 7
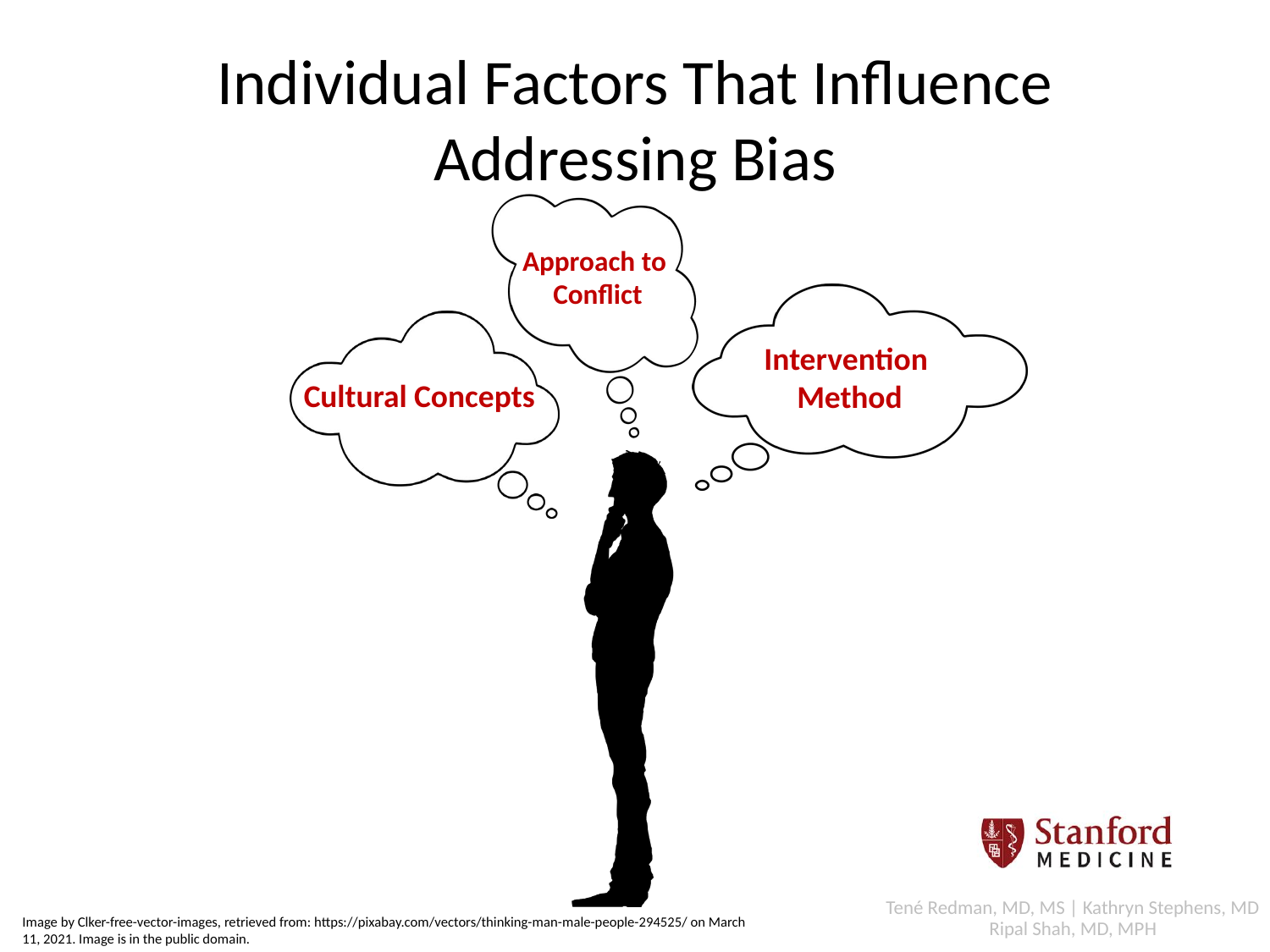

# Individual Factors That Influence Addressing Bias
Approach to
Conflict
Intervention
Method
Cultural Concepts
Tené Redman, MD, MS | Kathryn Stephens, MD
Ripal Shah, MD, MPH
Image by Clker-free-vector-images, retrieved from: https://pixabay.com/vectors/thinking-man-male-people-294525/ on March 11, 2021. Image is in the public domain.

## Slide 8
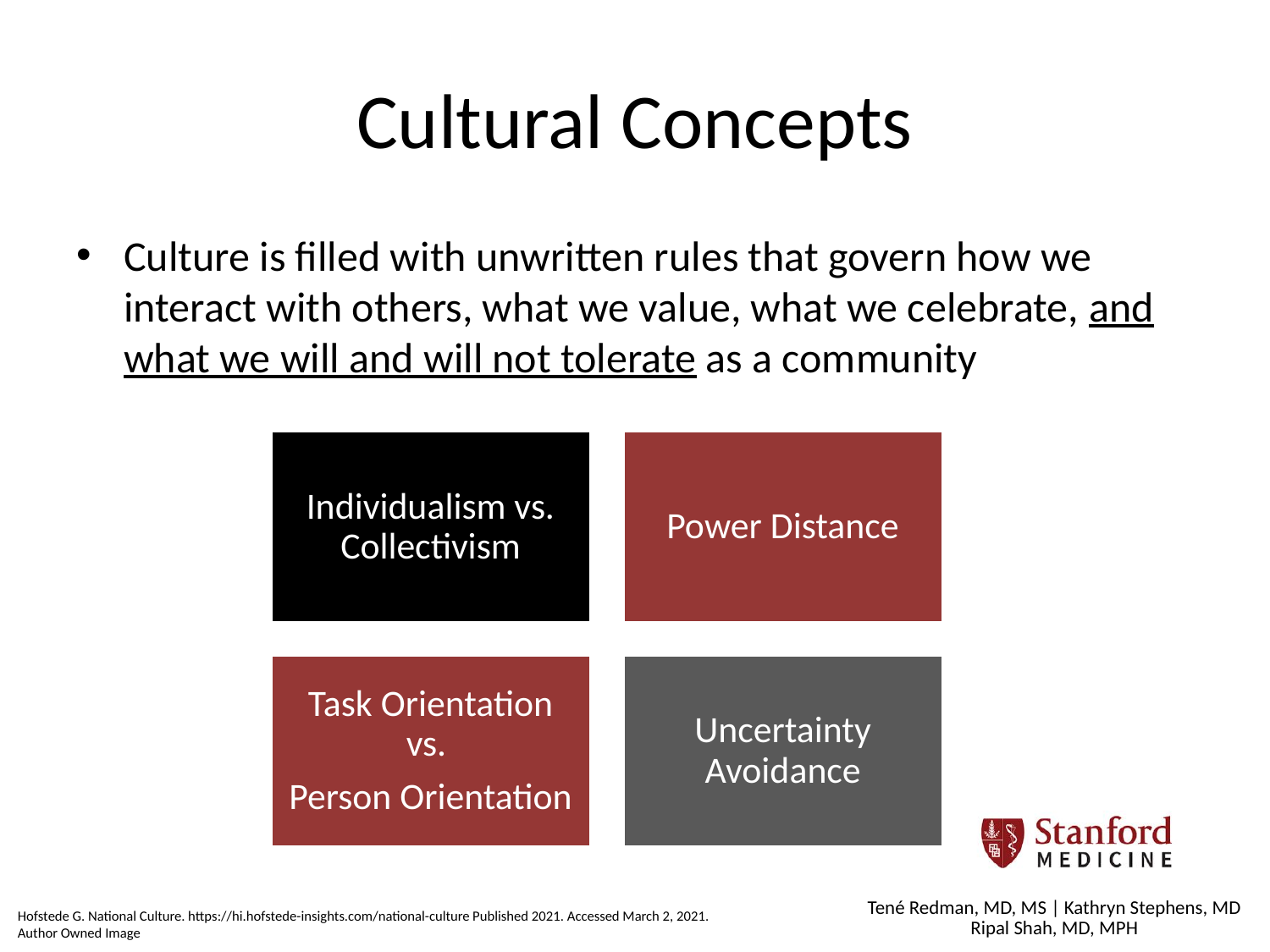

# Cultural Concepts
Culture is filled with unwritten rules that govern how we interact with others, what we value, what we celebrate, and what we will and will not tolerate as a community
Tené Redman, MD, MS | Kathryn Stephens, MD
Ripal Shah, MD, MPH
Hofstede G. National Culture. https://hi.hofstede-insights.com/national-culture Published 2021. Accessed March 2, 2021.
Author Owned Image

## Slide 9
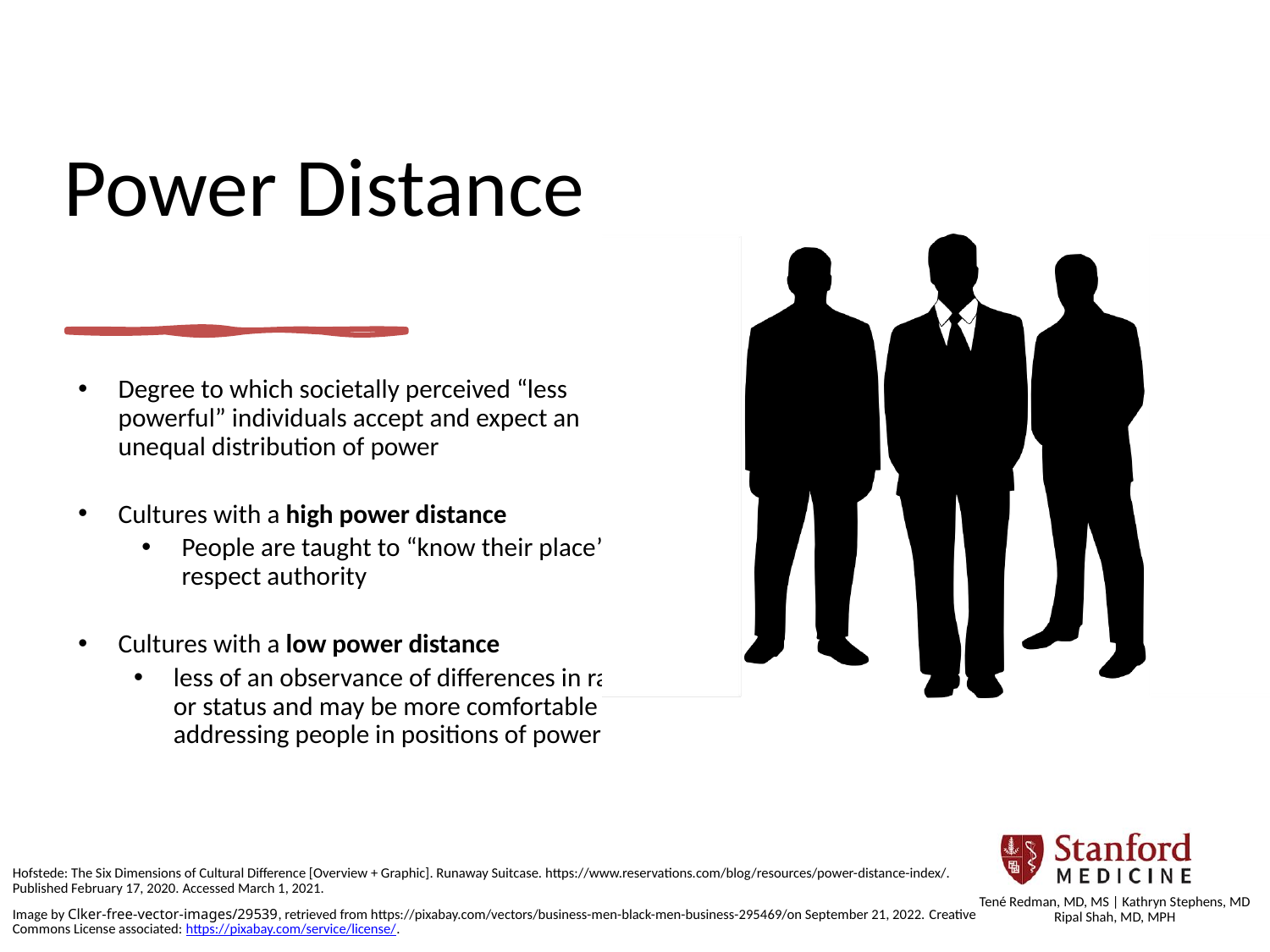

# Power Distance
Degree to which societally perceived “less powerful” individuals accept and expect an unequal distribution of power
Cultures with a high power distance
People are taught to “know their place” and respect authority
Cultures with a low power distance
less of an observance of differences in rank or status and may be more comfortable addressing people in positions of power
Hofstede: The Six Dimensions of Cultural Difference [Overview + Graphic]. Runaway Suitcase. https://www.reservations.com/blog/resources/power-distance-index/. Published February 17, 2020. Accessed March 1, 2021.
Image by Clker-free-vector-images/29539, retrieved from https://pixabay.com/vectors/business-men-black-men-business-295469/on September 21, 2022. Creative Commons License associated: https://pixabay.com/service/license/.
Tené Redman, MD, MS | Kathryn Stephens, MD
Ripal Shah, MD, MPH

## Slide 10
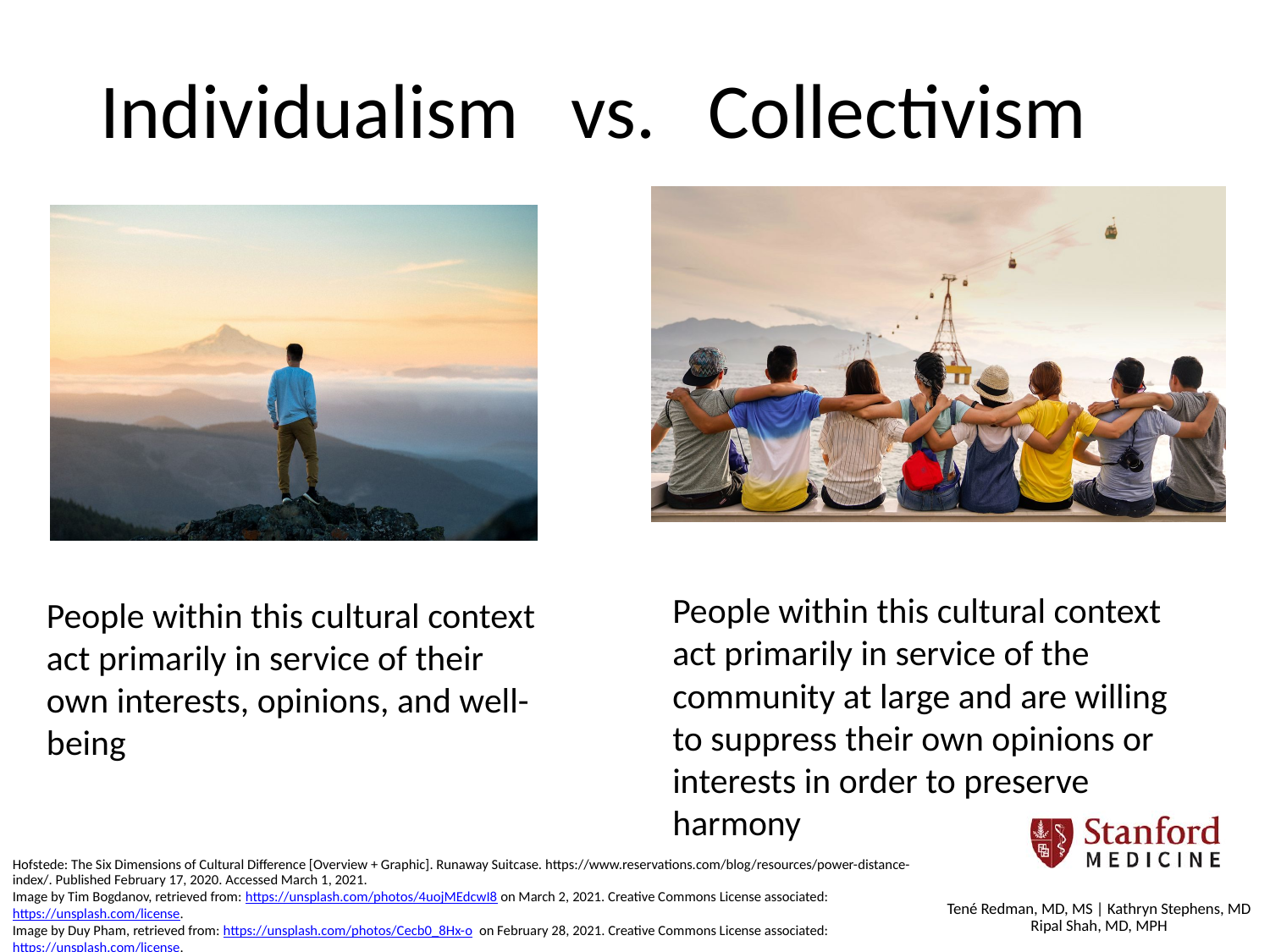

# Individualism vs. Collectivism
People within this cultural context act primarily in service of the community at large and are willing to suppress their own opinions or interests in order to preserve harmony
People within this cultural context act primarily in service of their own interests, opinions, and well-being
Hofstede: The Six Dimensions of Cultural Difference [Overview + Graphic]. Runaway Suitcase. https://www.reservations.com/blog/resources/power-distance-index/. Published February 17, 2020. Accessed March 1, 2021.
Image by Tim Bogdanov, retrieved from: https://unsplash.com/photos/4uojMEdcwI8 on March 2, 2021. Creative Commons License associated: https://unsplash.com/license.
Image by Duy Pham, retrieved from: https://unsplash.com/photos/Cecb0_8Hx-o  on February 28, 2021. Creative Commons License associated: https://unsplash.com/license.
Tené Redman, MD, MS | Kathryn Stephens, MD
Ripal Shah, MD, MPH

## Slide 11
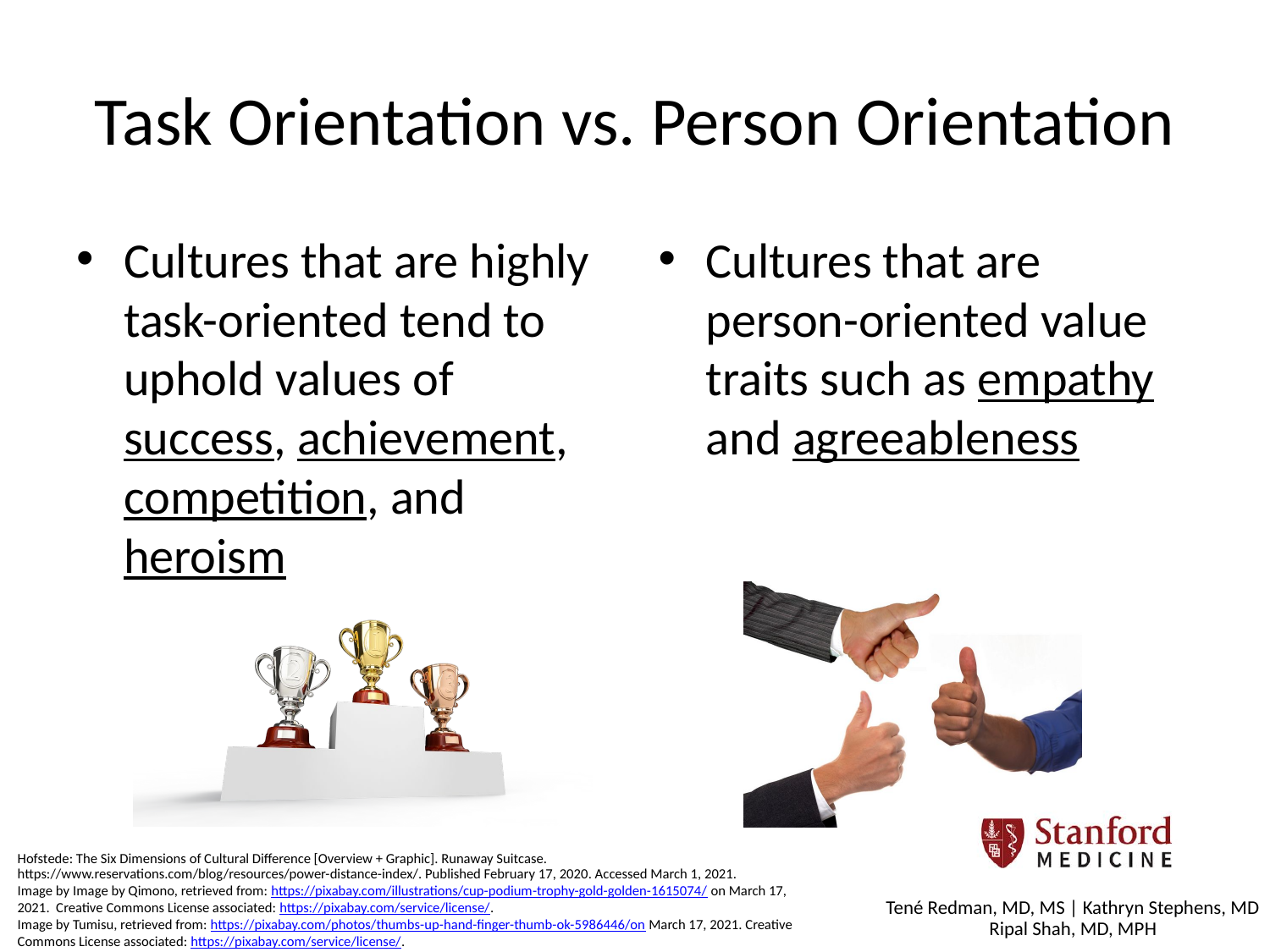

# Task Orientation vs. Person Orientation
Cultures that are highly task-oriented tend to uphold values of success, achievement, competition, and heroism
Cultures that are person-oriented value traits such as empathy and agreeableness
Hofstede: The Six Dimensions of Cultural Difference [Overview + Graphic]. Runaway Suitcase. https://www.reservations.com/blog/resources/power-distance-index/. Published February 17, 2020. Accessed March 1, 2021.
Image by Image by Qimono, retrieved from: https://pixabay.com/illustrations/cup-podium-trophy-gold-golden-1615074/ on March 17, 2021.  Creative Commons License associated: https://pixabay.com/service/license/.
Image by Tumisu, retrieved from: https://pixabay.com/photos/thumbs-up-hand-finger-thumb-ok-5986446/on March 17, 2021. Creative Commons License associated: https://pixabay.com/service/license/.
Tené Redman, MD, MS | Kathryn Stephens, MD
Ripal Shah, MD, MPH

## Slide 12
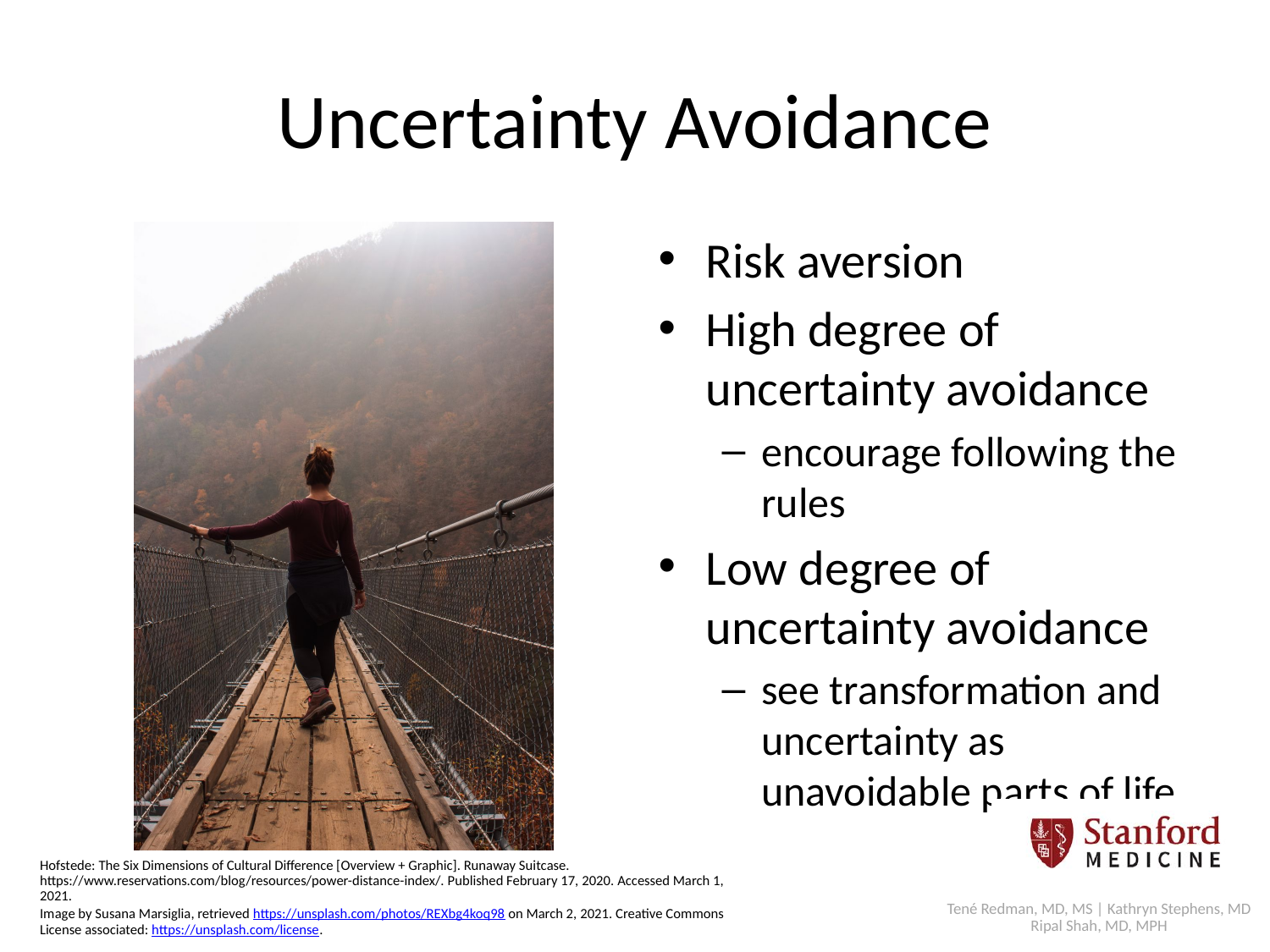

# Uncertainty Avoidance
Risk aversion
High degree of uncertainty avoidance
encourage following the rules
Low degree of uncertainty avoidance
see transformation and uncertainty as unavoidable parts of life
Hofstede: The Six Dimensions of Cultural Difference [Overview + Graphic]. Runaway Suitcase. https://www.reservations.com/blog/resources/power-distance-index/. Published February 17, 2020. Accessed March 1, 2021.
Image by Susana Marsiglia, retrieved https://unsplash.com/photos/REXbg4koq98 on March 2, 2021. Creative Commons License associated: https://unsplash.com/license.
Tené Redman, MD, MS | Kathryn Stephens, MD
Ripal Shah, MD, MPH

## Slide 13
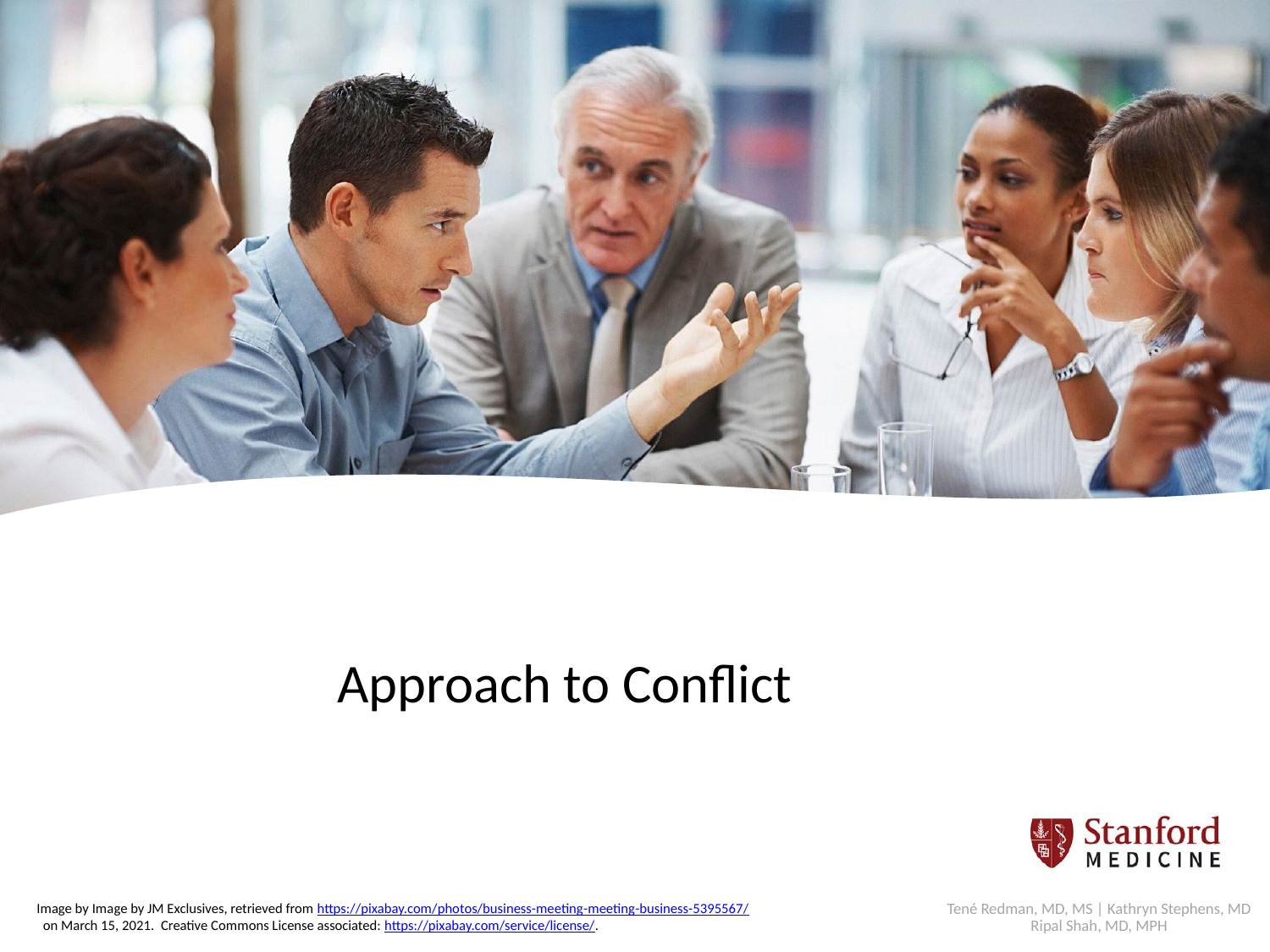

# Approach to Conflict
Image by Image by JM Exclusives, retrieved from https://pixabay.com/photos/business-meeting-meeting-business-5395567/  on March 15, 2021.  Creative Commons License associated: https://pixabay.com/service/license/.
Tené Redman, MD, MS | Kathryn Stephens, MD
Ripal Shah, MD, MPH

## Slide 14
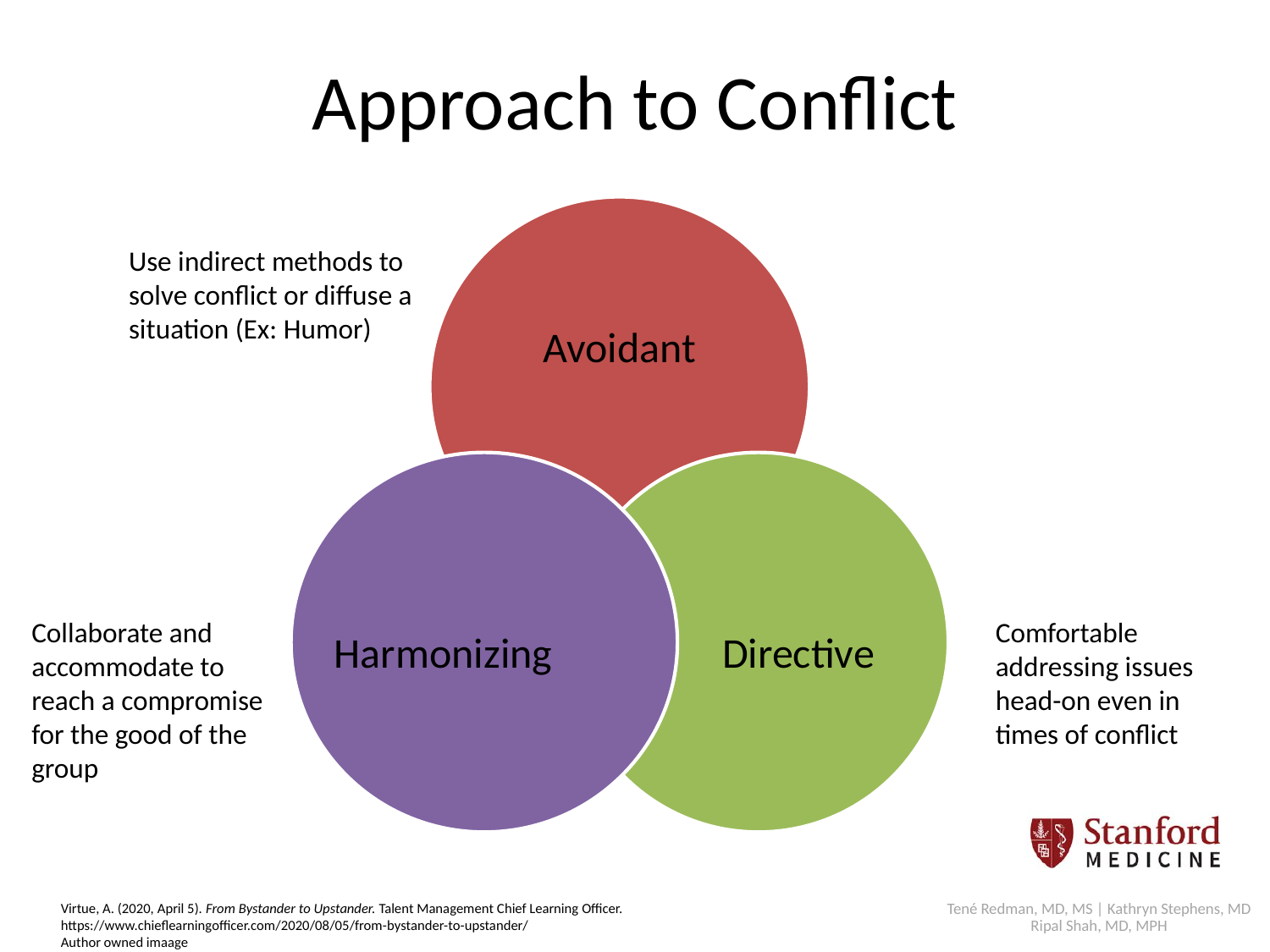

# Approach to Conflict
Avoidant
Harmonizing
Directive
Use indirect methods to solve conflict or diffuse a situation (Ex: Humor)
Collaborate and accommodate to reach a compromise for the good of the group
Comfortable addressing issues head-on even in times of conflict
Virtue, A. (2020, April 5). From Bystander to Upstander. Talent Management Chief Learning Officer. https://www.chieflearningofficer.com/2020/08/05/from-bystander-to-upstander/
Author owned imaage
Tené Redman, MD, MS | Kathryn Stephens, MD
Ripal Shah, MD, MPH

## Slide 15
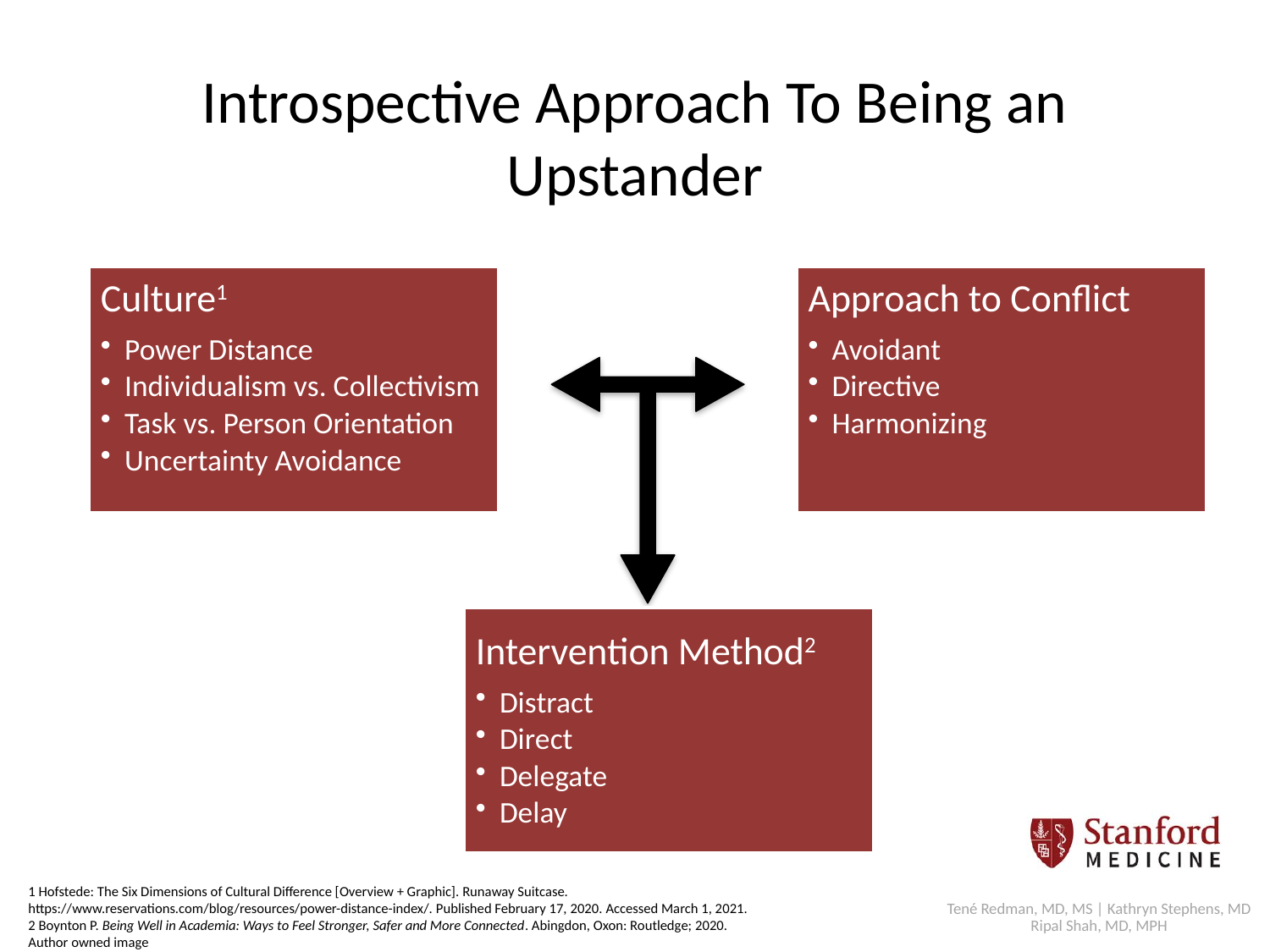

# Introspective Approach To Being an Upstander
Approach to Conflict
Avoidant
Directive
Harmonizing
Culture1
Power Distance
Individualism vs. Collectivism
Task vs. Person Orientation
Uncertainty Avoidance
Intervention Method2
Distract
Direct
Delegate
Delay
1 Hofstede: The Six Dimensions of Cultural Difference [Overview + Graphic]. Runaway Suitcase. https://www.reservations.com/blog/resources/power-distance-index/. Published February 17, 2020. Accessed March 1, 2021.
2 Boynton P. Being Well in Academia: Ways to Feel Stronger, Safer and More Connected. Abingdon, Oxon: Routledge; 2020.
Author owned image
Tené Redman, MD, MS | Kathryn Stephens, MD
Ripal Shah, MD, MPH

## Slide 16
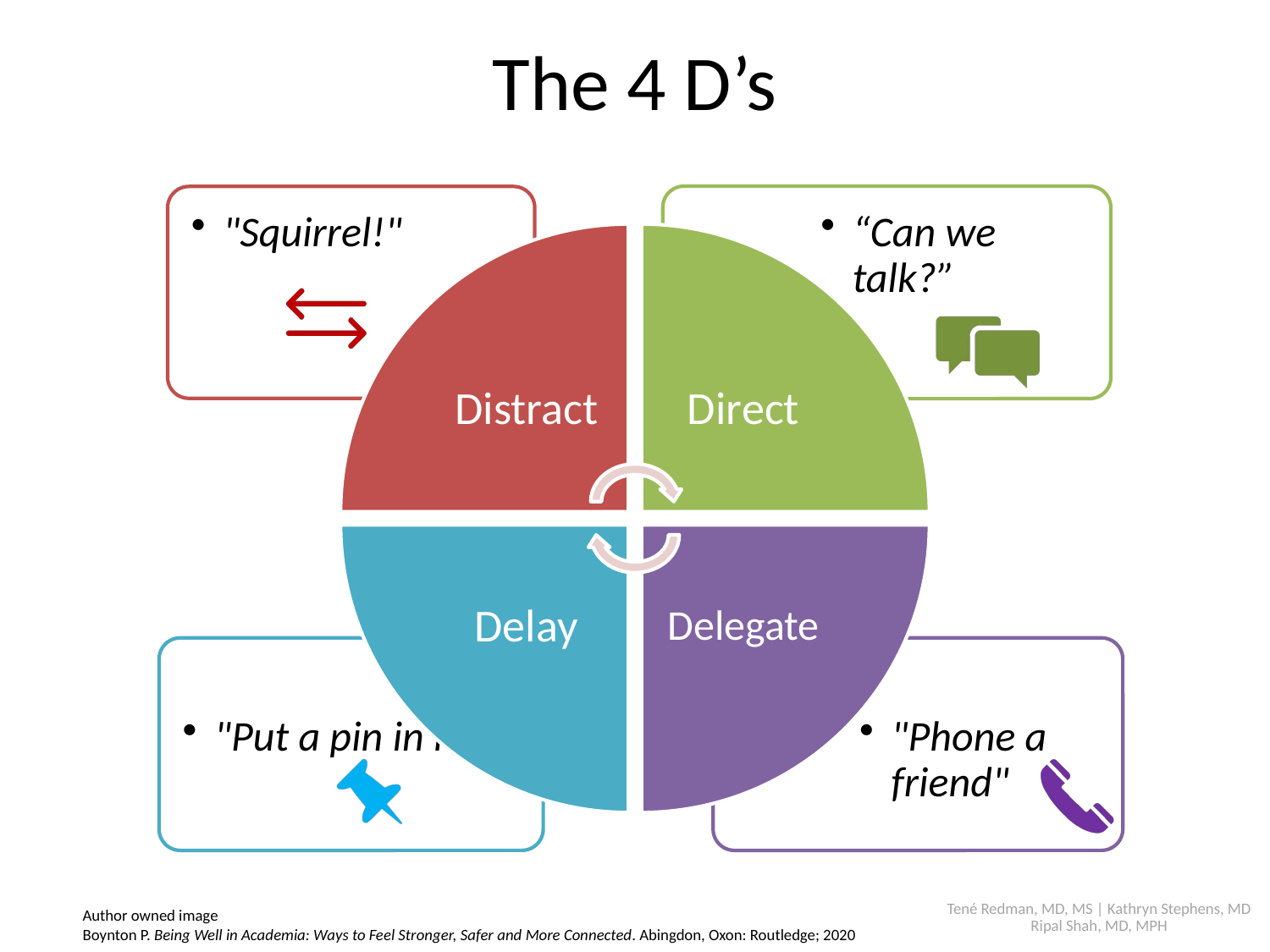

# The 4 D’s
Tené Redman, MD, MS | Kathryn Stephens, MD
Ripal Shah, MD, MPH
Author owned image
Boynton P. Being Well in Academia: Ways to Feel Stronger, Safer and More Connected. Abingdon, Oxon: Routledge; 2020

## Slide 17
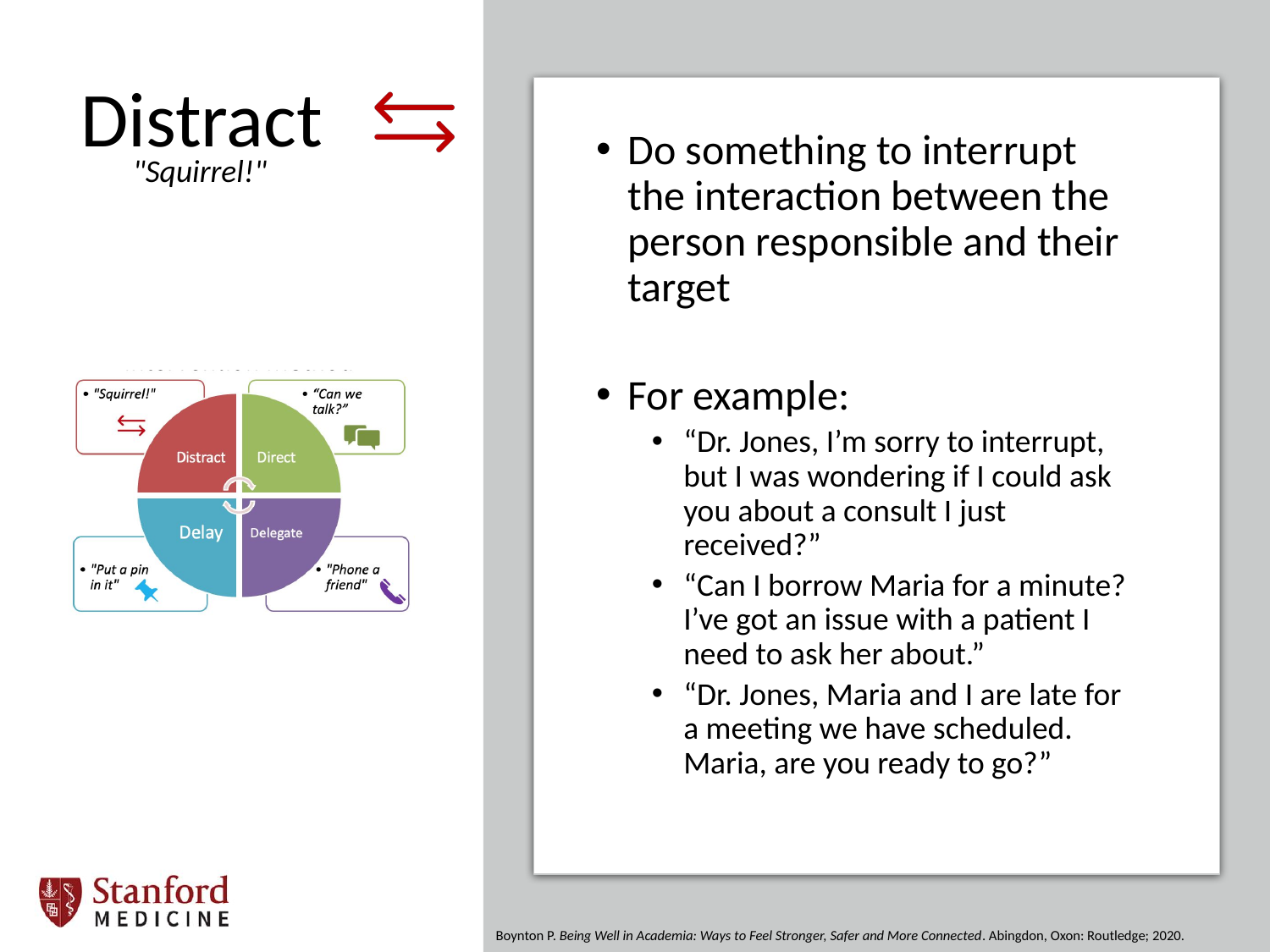

# Distract
Do something to interrupt the interaction between the person responsible and their target
For example:
“Dr. Jones, I’m sorry to interrupt, but I was wondering if I could ask you about a consult I just received?”
“Can I borrow Maria for a minute? I’ve got an issue with a patient I need to ask her about.”
“Dr. Jones, Maria and I are late for a meeting we have scheduled. Maria, are you ready to go?”
"Squirrel!"
Boynton P. Being Well in Academia: Ways to Feel Stronger, Safer and More Connected. Abingdon, Oxon: Routledge; 2020.

## Slide 18
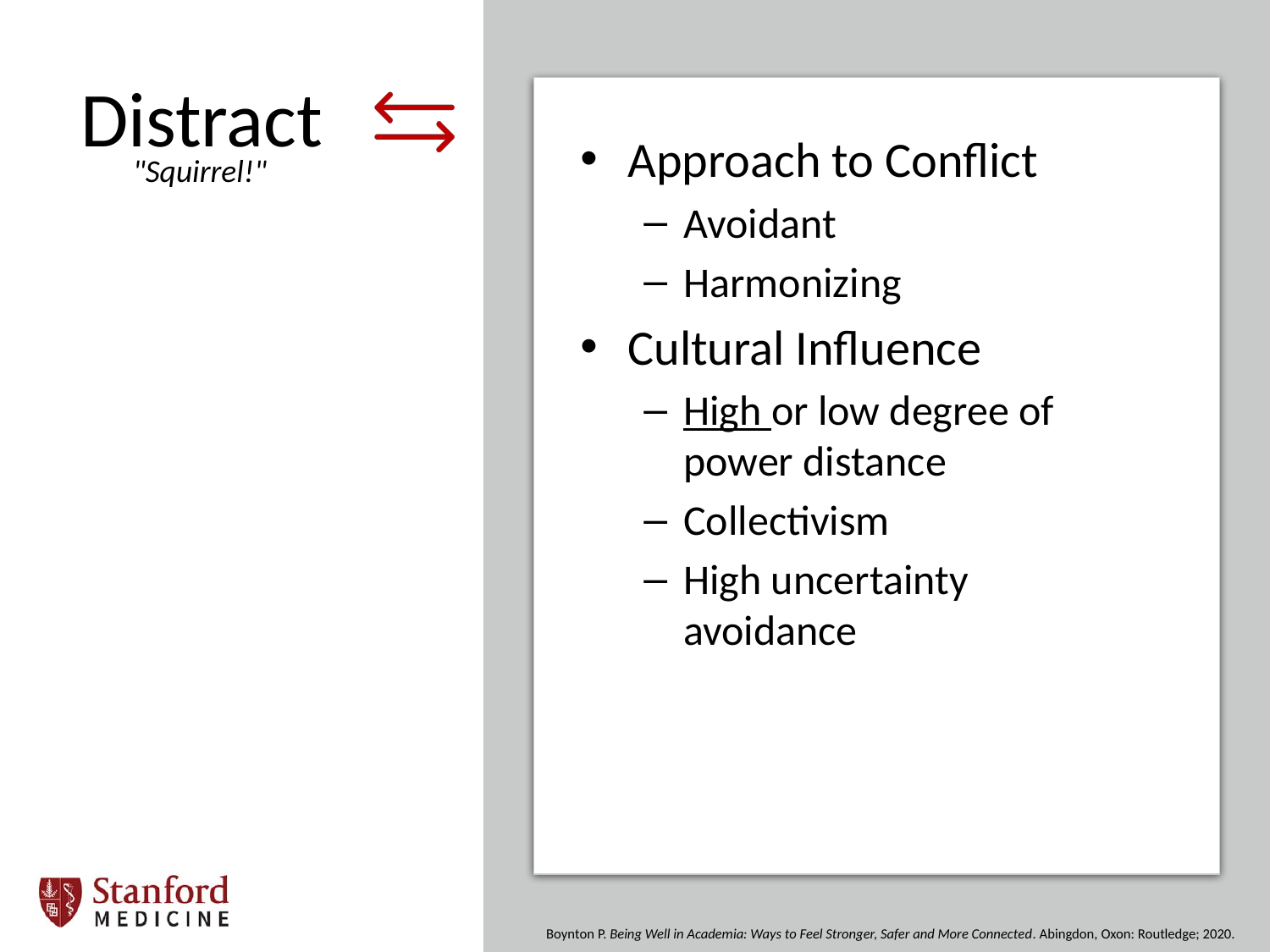

# Distract
Approach to Conflict
Avoidant
Harmonizing
Cultural Influence
High or low degree of power distance
Collectivism
High uncertainty avoidance
"Squirrel!"
Boynton P. Being Well in Academia: Ways to Feel Stronger, Safer and More Connected. Abingdon, Oxon: Routledge; 2020.

## Slide 19
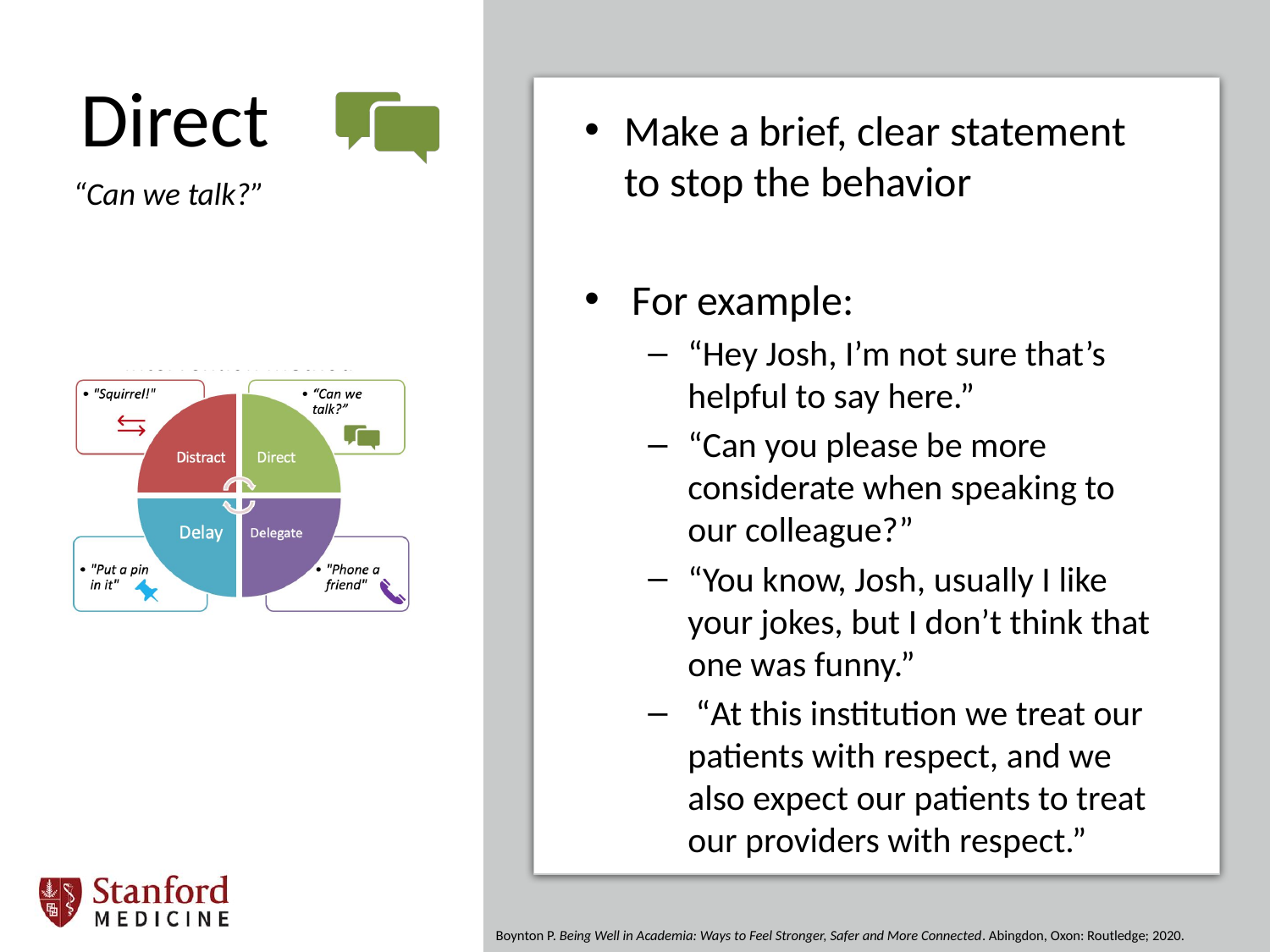

# Direct
Make a brief, clear statement to stop the behavior
For example:
“Hey Josh, I’m not sure that’s helpful to say here.”
“Can you please be more considerate when speaking to our colleague?”
“You know, Josh, usually I like your jokes, but I don’t think that one was funny.”
 “At this institution we treat our patients with respect, and we also expect our patients to treat our providers with respect.”
“Can we talk?”
Boynton P. Being Well in Academia: Ways to Feel Stronger, Safer and More Connected. Abingdon, Oxon: Routledge; 2020.

## Slide 20
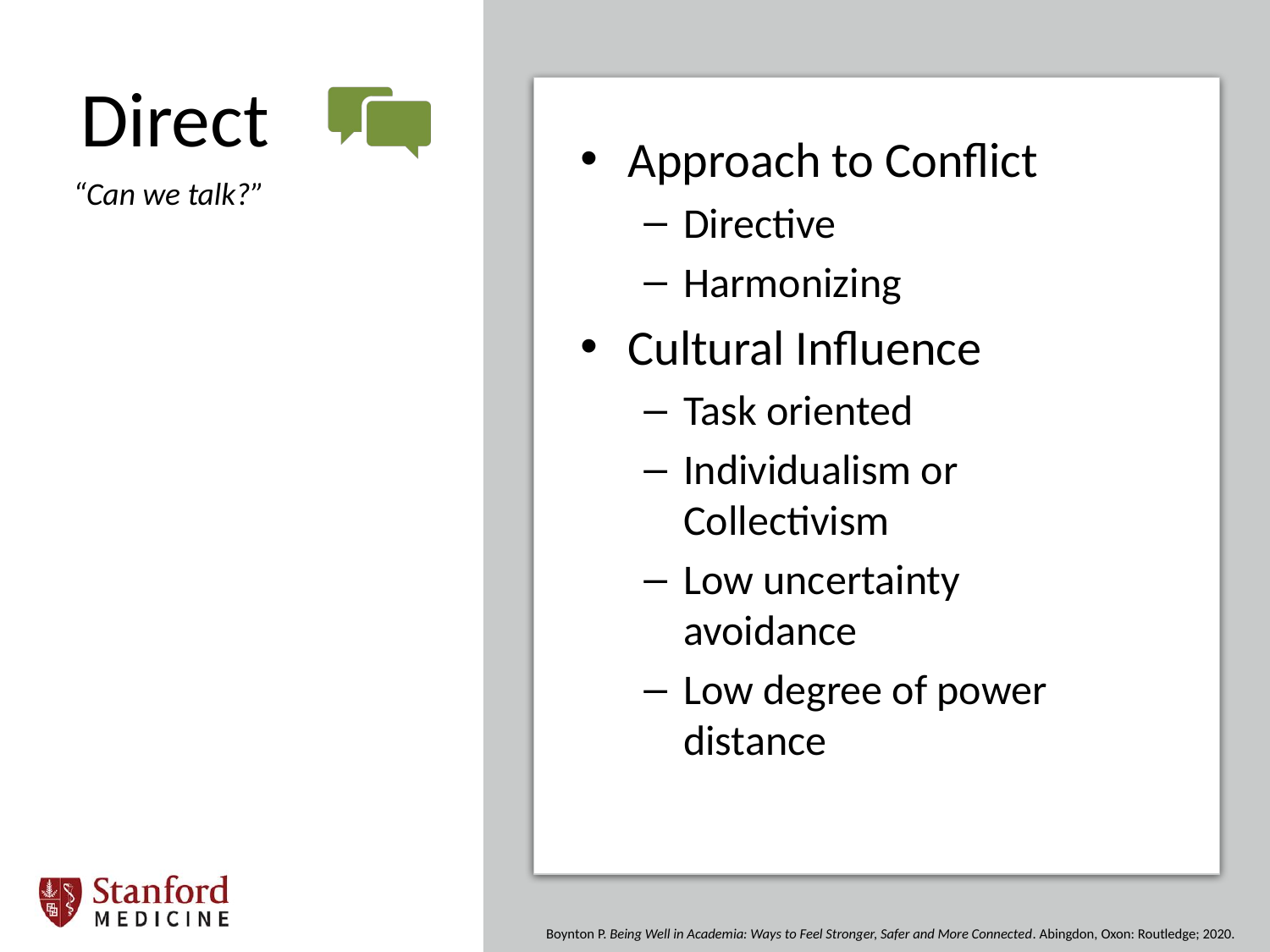

# Direct
Approach to Conflict
Directive
Harmonizing
Cultural Influence
Task oriented
Individualism or Collectivism
Low uncertainty avoidance
Low degree of power distance
“Can we talk?”
Boynton P. Being Well in Academia: Ways to Feel Stronger, Safer and More Connected. Abingdon, Oxon: Routledge; 2020.

## Slide 21
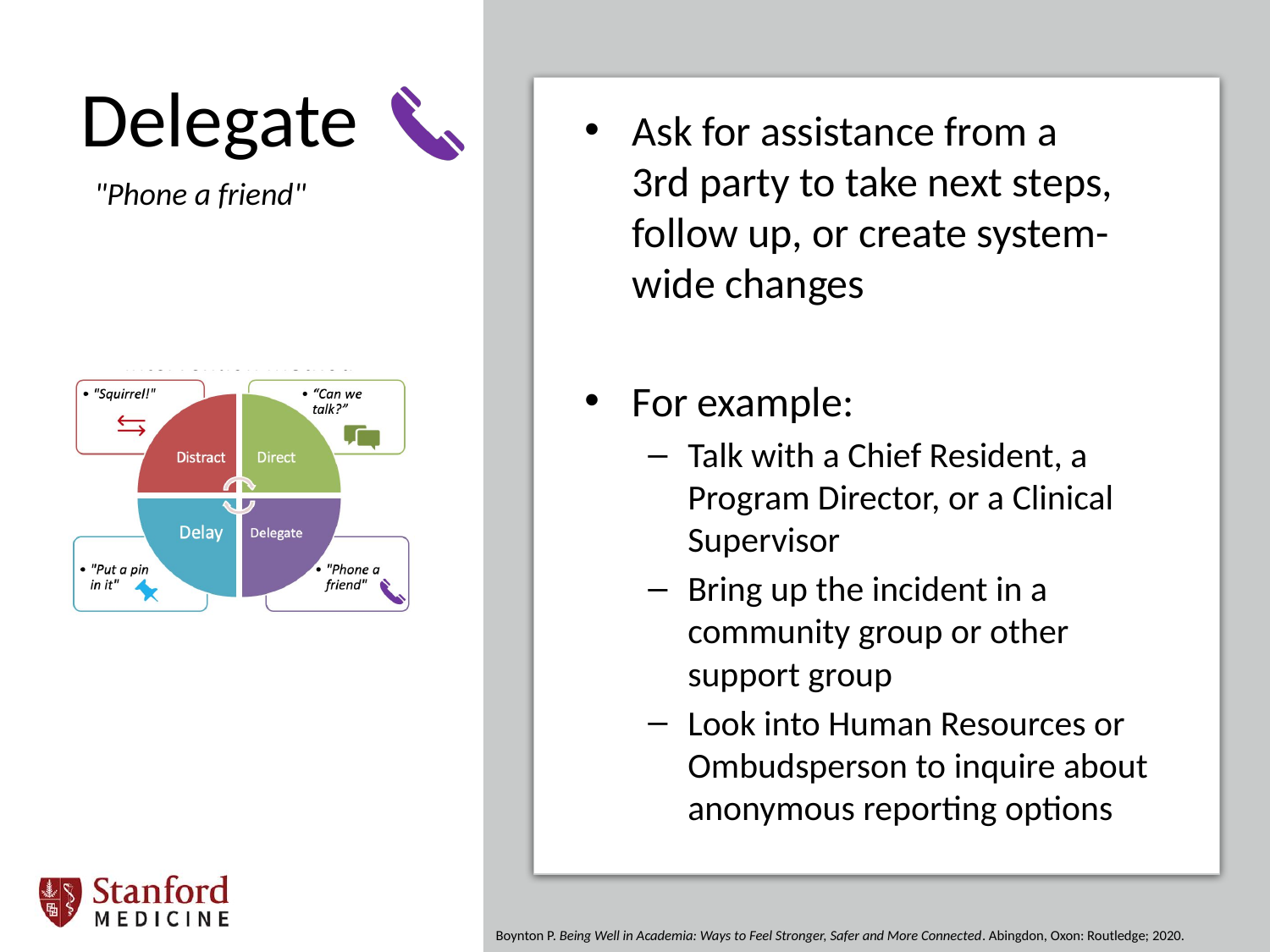

# Delegate
Ask for assistance from a 3rd party to take next steps, follow up, or create system-wide changes​
For example: ​
Talk with a Chief Resident, a Program Director, or a Clinical Supervisor  ​
Bring up the incident in a community group or other support group​
Look into Human Resources or Ombudsperson to inquire about anonymous reporting options ​
"Phone a friend"
Boynton P. Being Well in Academia: Ways to Feel Stronger, Safer and More Connected. Abingdon, Oxon: Routledge; 2020.

## Slide 22
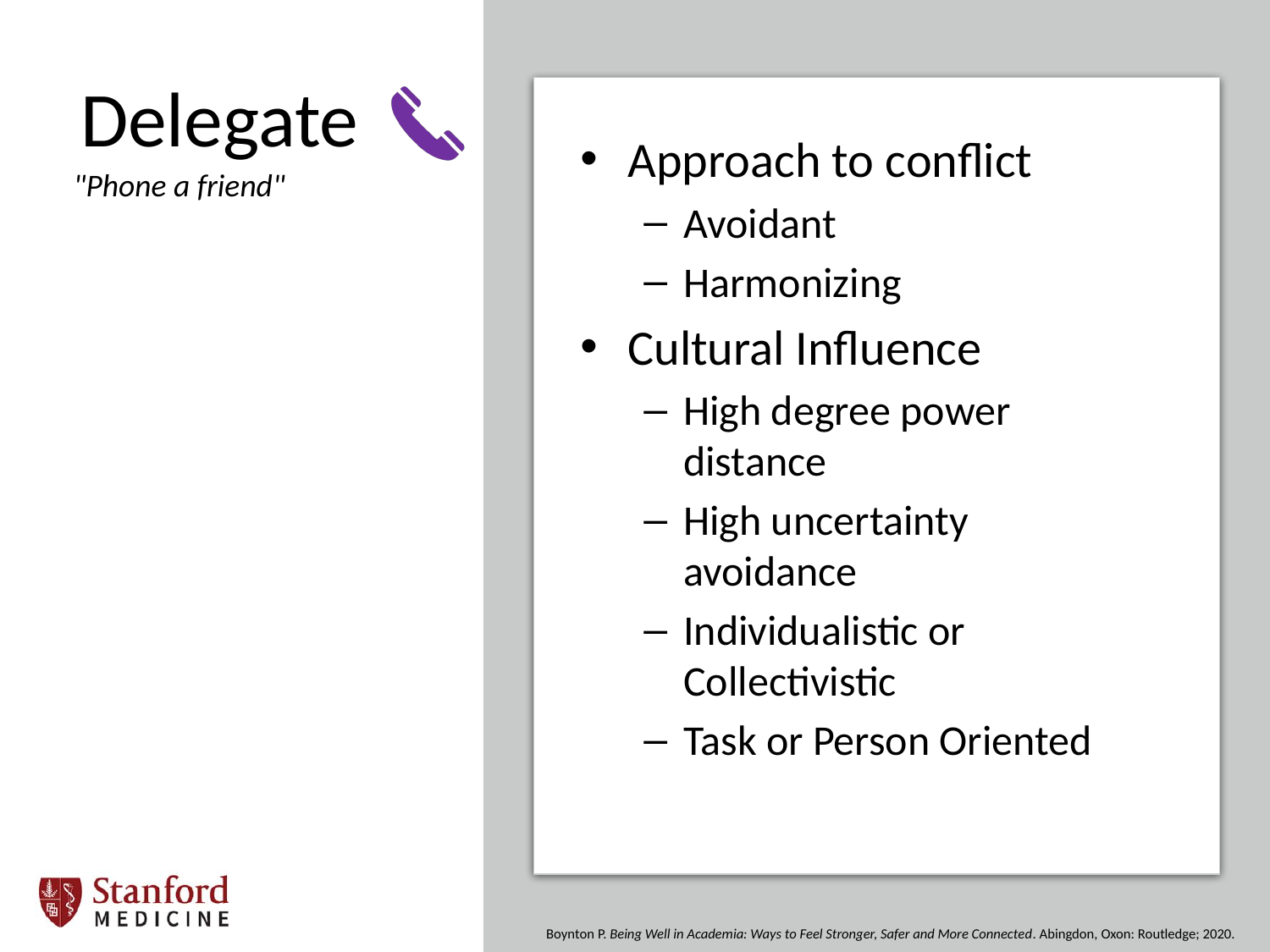

# Delegate
Approach to conflict
Avoidant
Harmonizing
Cultural Influence
High degree power distance
High uncertainty avoidance
Individualistic or Collectivistic
Task or Person Oriented
"Phone a friend"
Boynton P. Being Well in Academia: Ways to Feel Stronger, Safer and More Connected. Abingdon, Oxon: Routledge; 2020.

## Slide 23
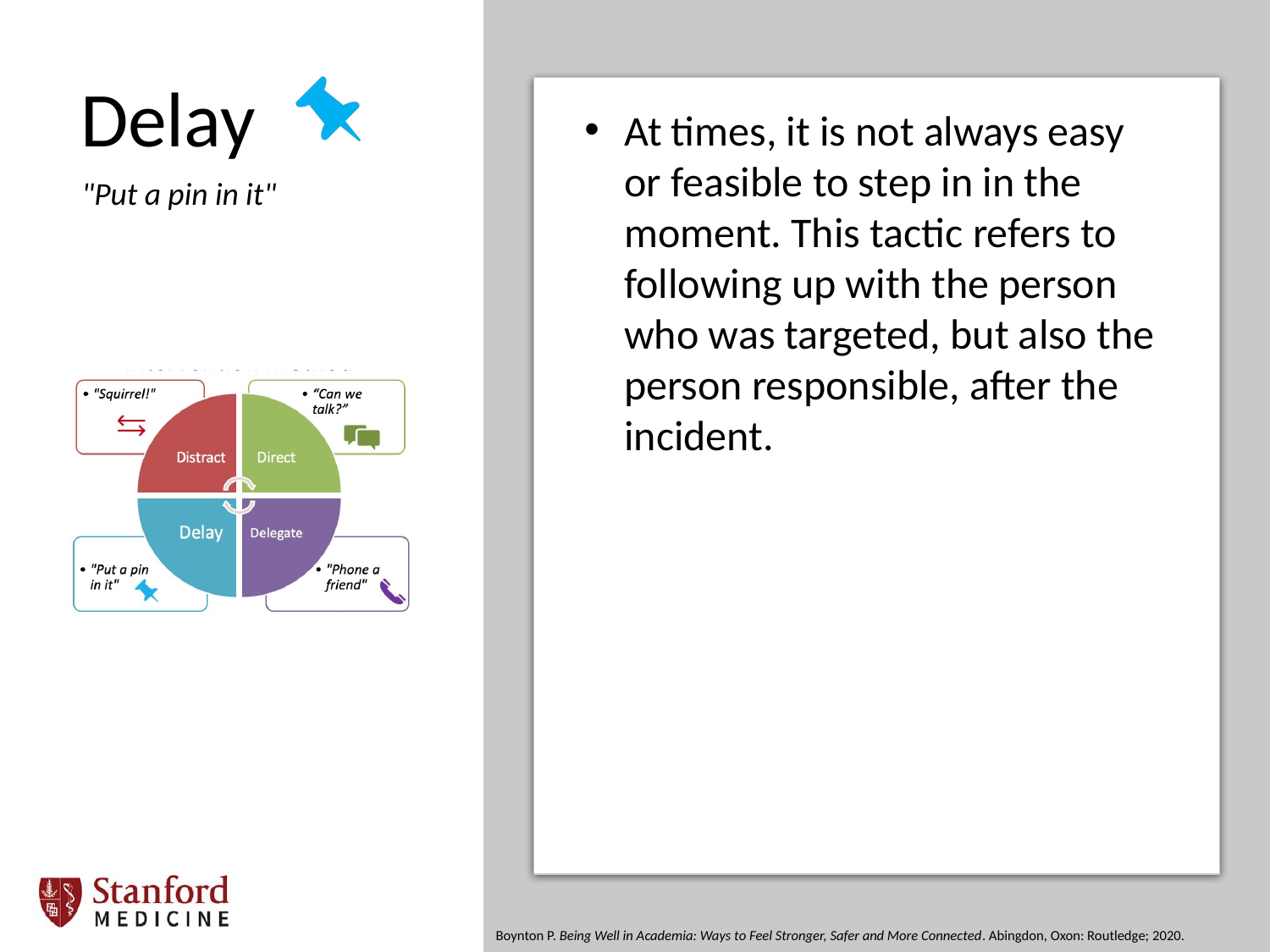

# Delay
At times, it is not always easy or feasible to step in in the moment. This tactic refers to following up with the person who was targeted, but also the person responsible, after the incident.
"Put a pin in it"
Boynton P. Being Well in Academia: Ways to Feel Stronger, Safer and More Connected. Abingdon, Oxon: Routledge; 2020.

## Slide 24
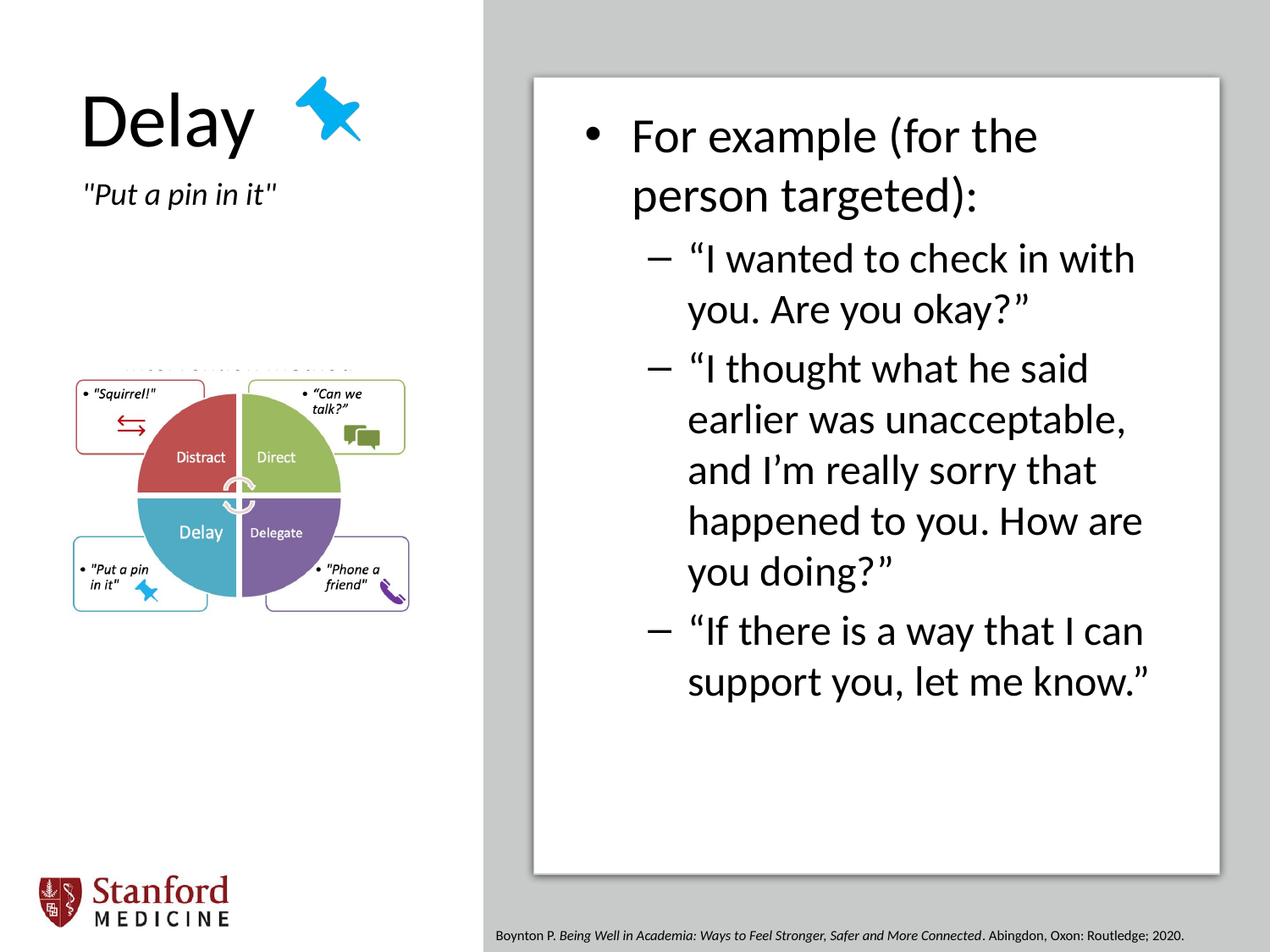

# Delay
For example (for the person targeted):
“I wanted to check in with you. Are you okay?”
“I thought what he said earlier was unacceptable, and I’m really sorry that happened to you. How are you doing?”
“If there is a way that I can support you, let me know.”
"Put a pin in it"
Boynton P. Being Well in Academia: Ways to Feel Stronger, Safer and More Connected. Abingdon, Oxon: Routledge; 2020.

## Slide 25
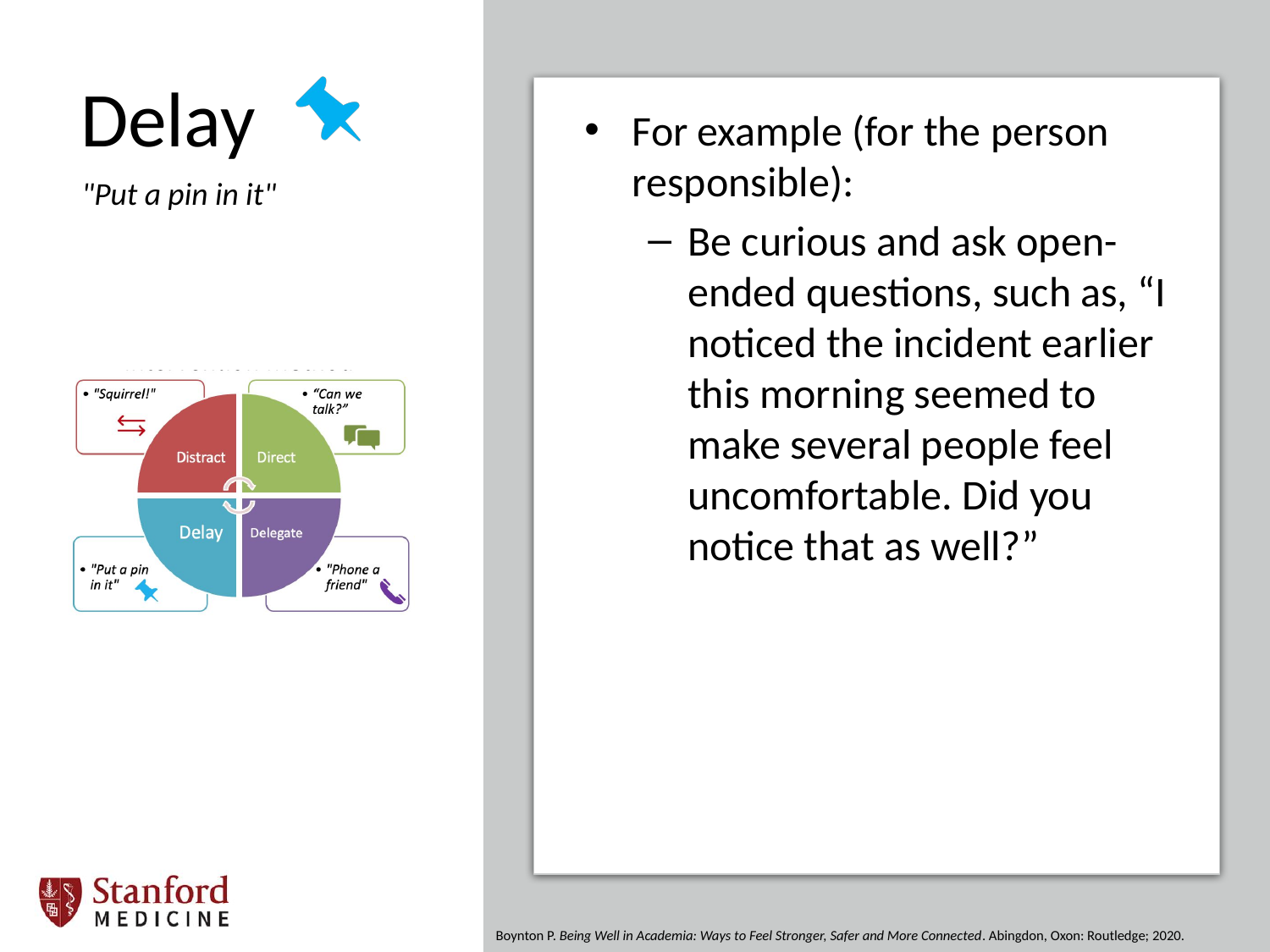

# Delay
For example (for the person responsible):
Be curious and ask open-ended questions, such as, “I noticed the incident earlier this morning seemed to make several people feel uncomfortable. Did you notice that as well?”
"Put a pin in it"
Boynton P. Being Well in Academia: Ways to Feel Stronger, Safer and More Connected. Abingdon, Oxon: Routledge; 2020.

## Slide 26
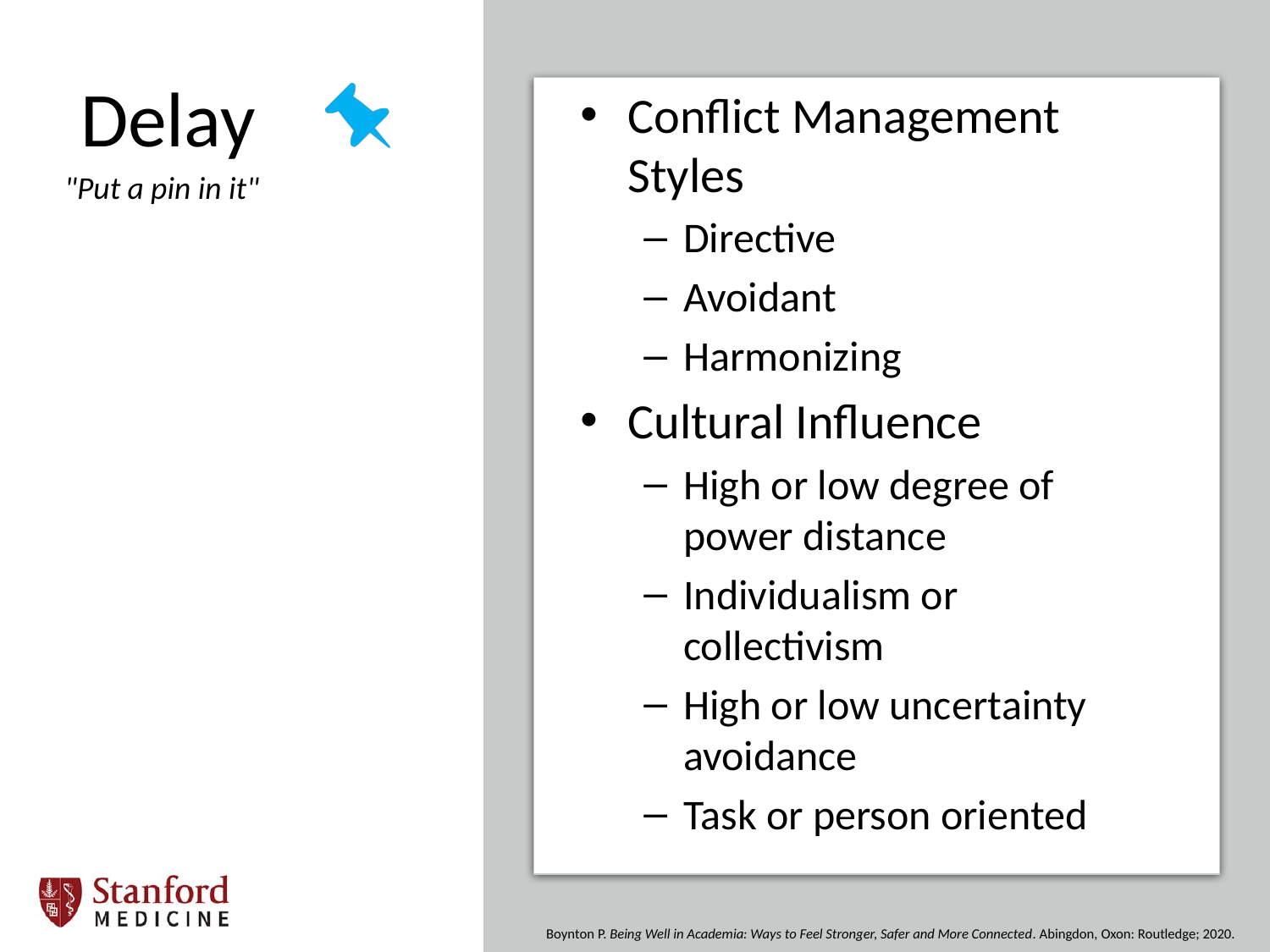

# Delay
Conflict Management Styles
Directive
Avoidant
Harmonizing
Cultural Influence
High or low degree of power distance
Individualism or collectivism
High or low uncertainty avoidance
Task or person oriented
"Put a pin in it"
Boynton P. Being Well in Academia: Ways to Feel Stronger, Safer and More Connected. Abingdon, Oxon: Routledge; 2020.

## Slide 27
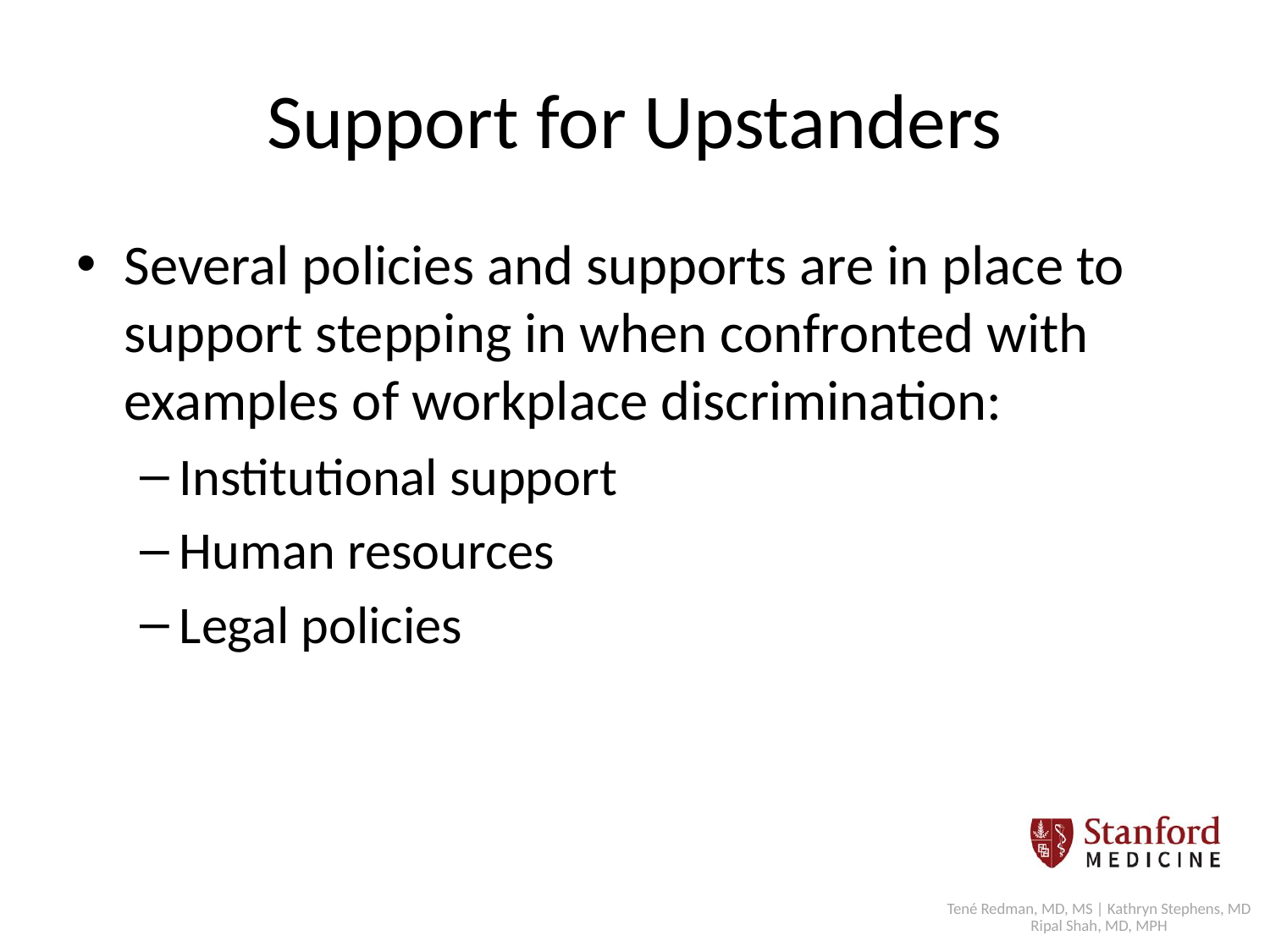

# Support for Upstanders
Several policies and supports are in place to support stepping in when confronted with examples of workplace discrimination:
Institutional support
Human resources
Legal policies
Tené Redman, MD, MS | Kathryn Stephens, MD
Ripal Shah, MD, MPH

## Slide 28
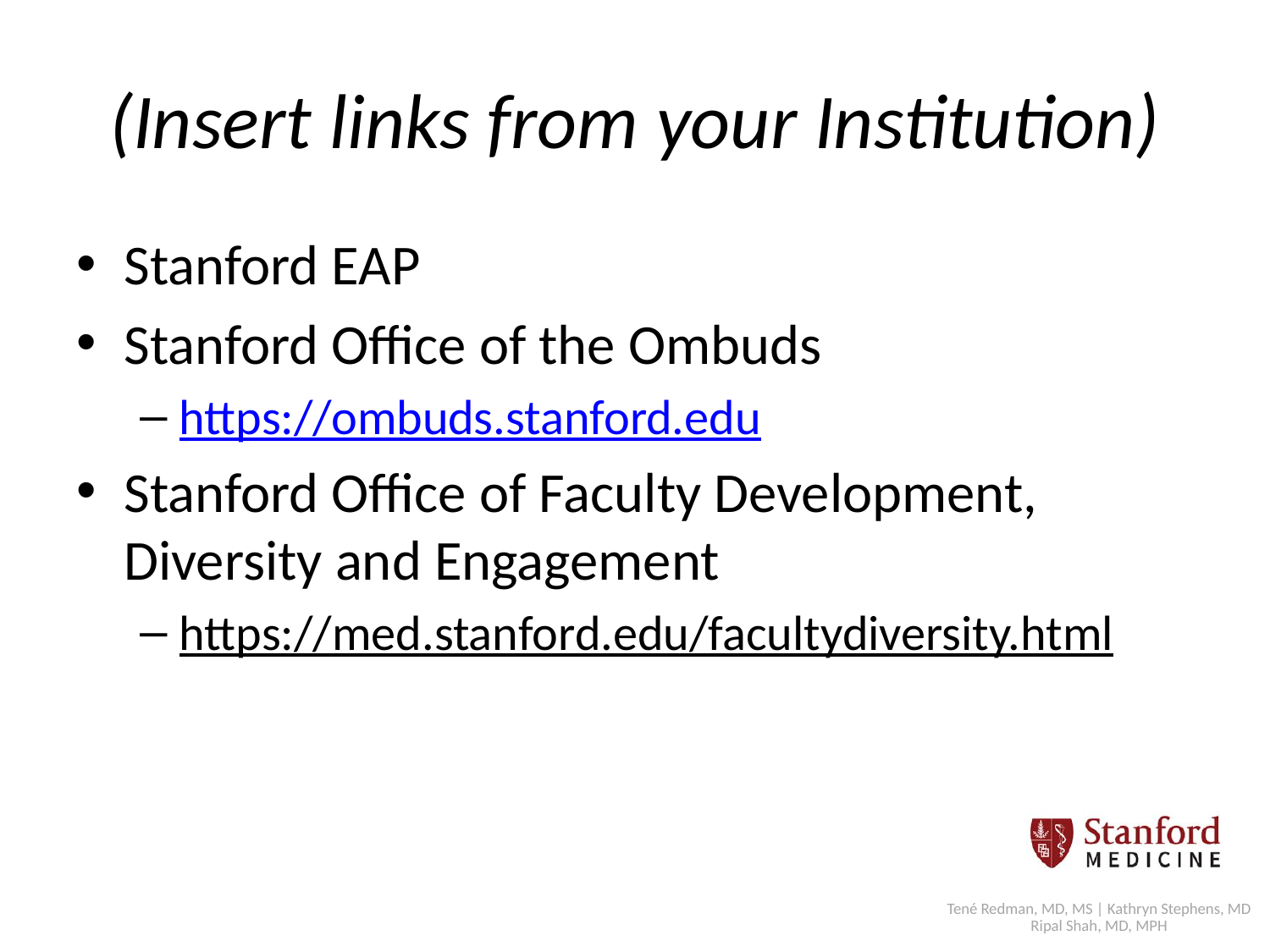

# (Insert links from your Institution)
Stanford EAP
Stanford Office of the Ombuds
https://ombuds.stanford.edu
Stanford Office of Faculty Development, Diversity and Engagement
https://med.stanford.edu/facultydiversity.html
Tené Redman, MD, MS | Kathryn Stephens, MD
Ripal Shah, MD, MPH

## Slide 29
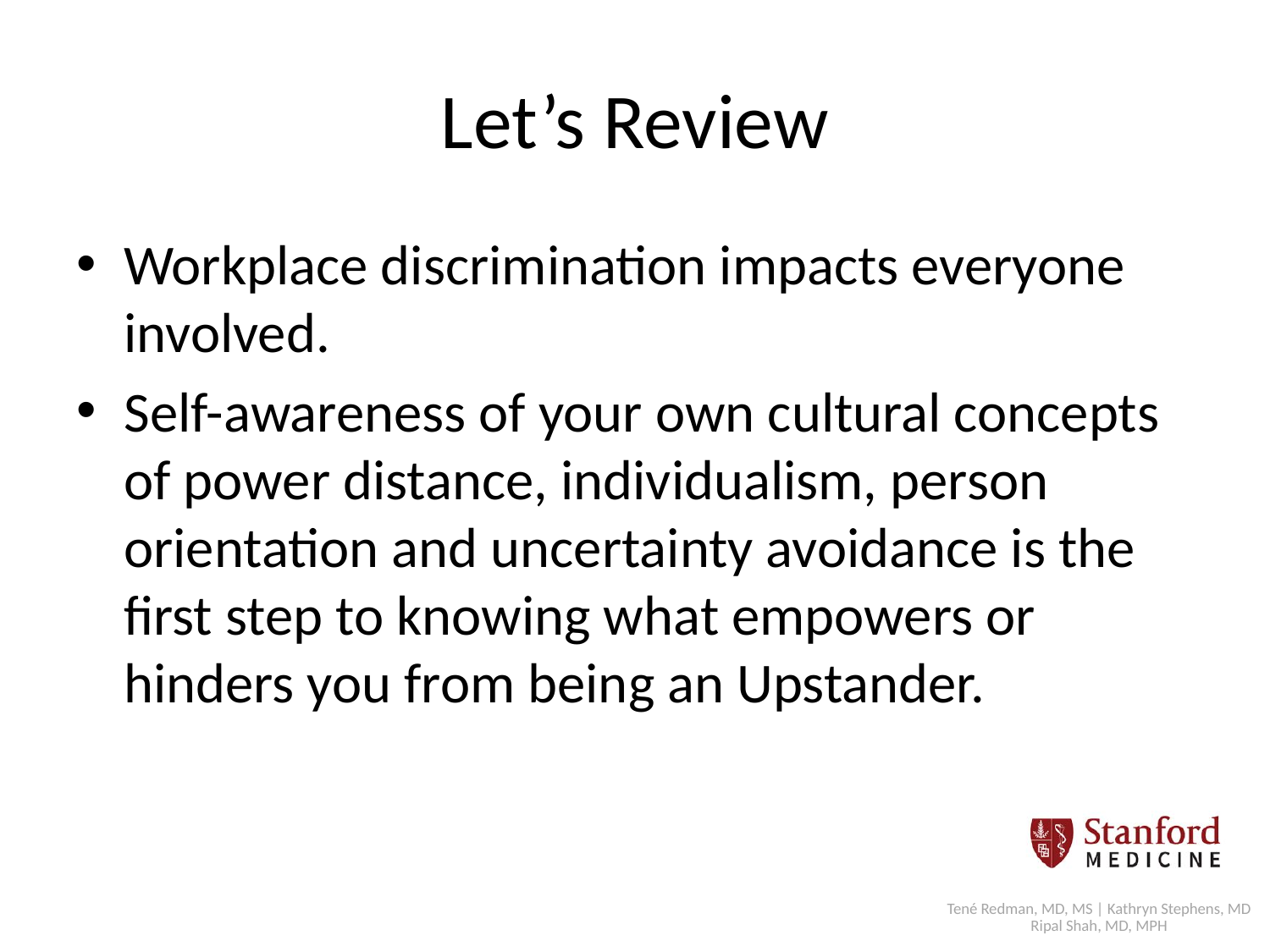

# Let’s Review
Workplace discrimination impacts everyone involved.
Self-awareness of your own cultural concepts of power distance, individualism, person orientation and uncertainty avoidance is the first step to knowing what empowers or hinders you from being an Upstander.
Tené Redman, MD, MS | Kathryn Stephens, MD
Ripal Shah, MD, MPH

## Slide 30
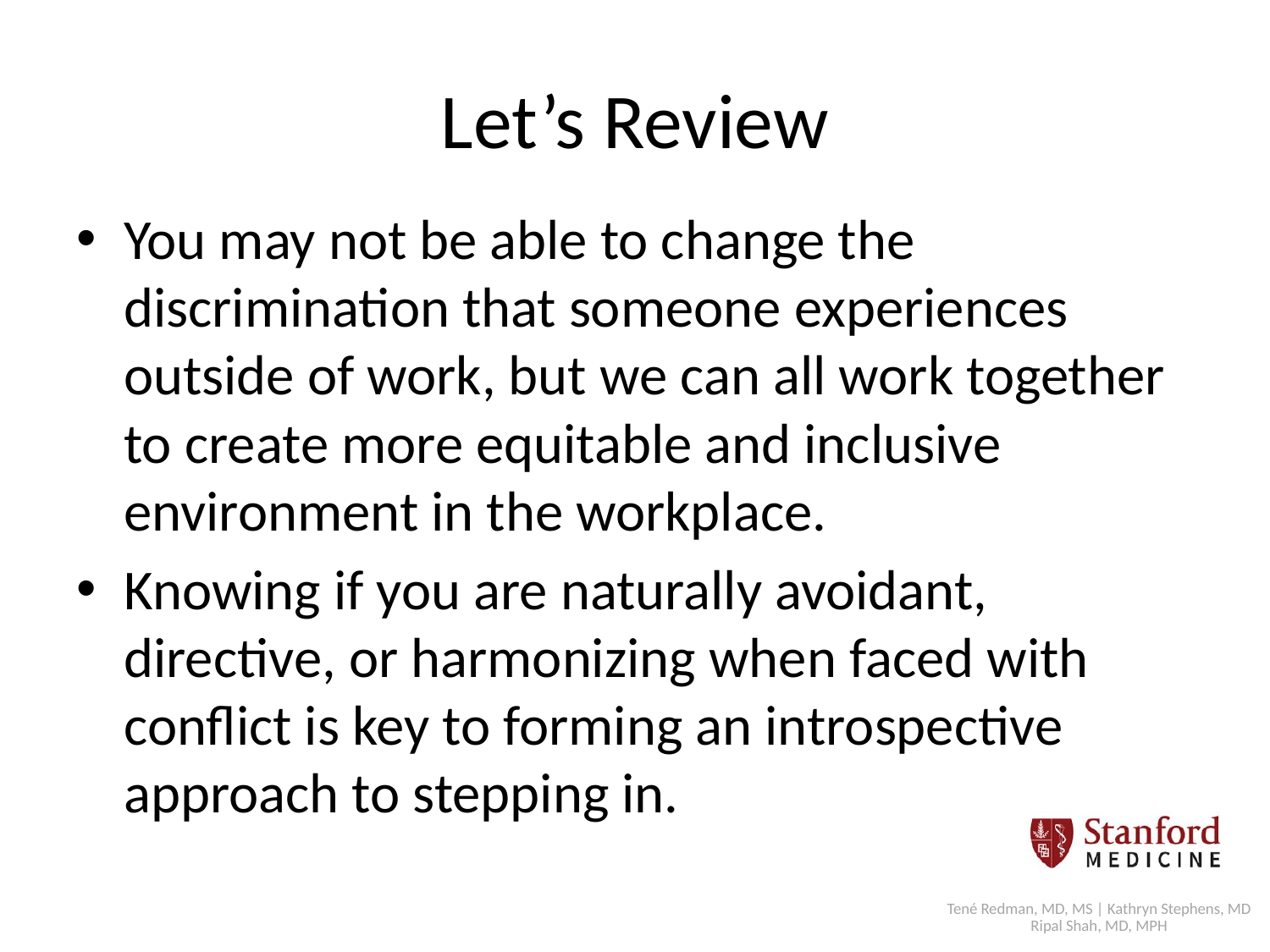

# Let’s Review
You may not be able to change the discrimination that someone experiences outside of work, but we can all work together to create more equitable and inclusive environment in the workplace.
Knowing if you are naturally avoidant, directive, or harmonizing when faced with conflict is key to forming an introspective approach to stepping in.
Tené Redman, MD, MS | Kathryn Stephens, MD
Ripal Shah, MD, MPH

## Slide 31
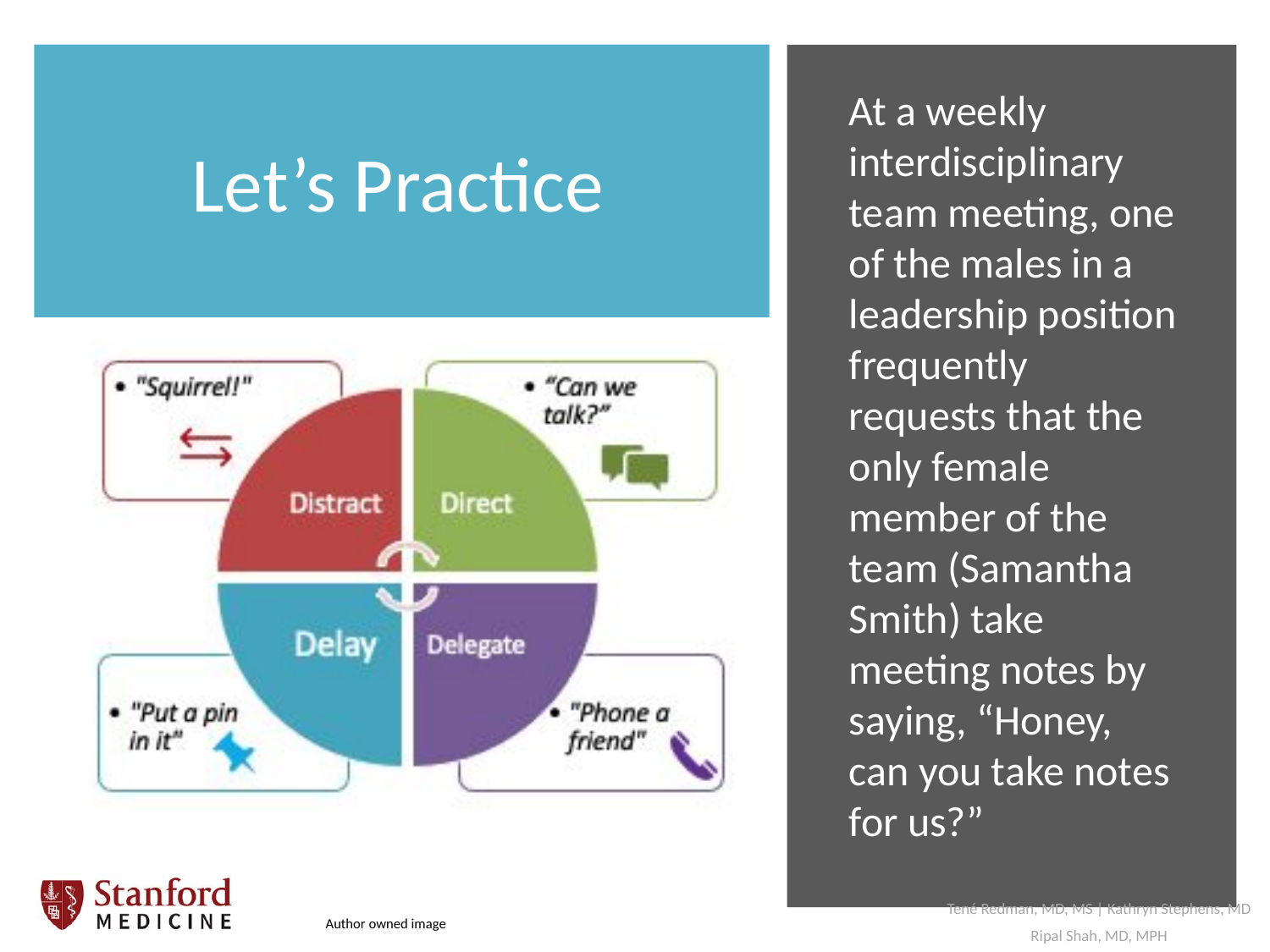

# Let’s Practice
At a weekly interdisciplinary team meeting, one of the males in a leadership position frequently requests that the only female member of the team (Samantha Smith) take meeting notes by saying, “Honey, can you take notes for us?”
Tené Redman, MD, MS | Kathryn Stephens, MD
Ripal Shah, MD, MPH
Author owned image

## Slide 32
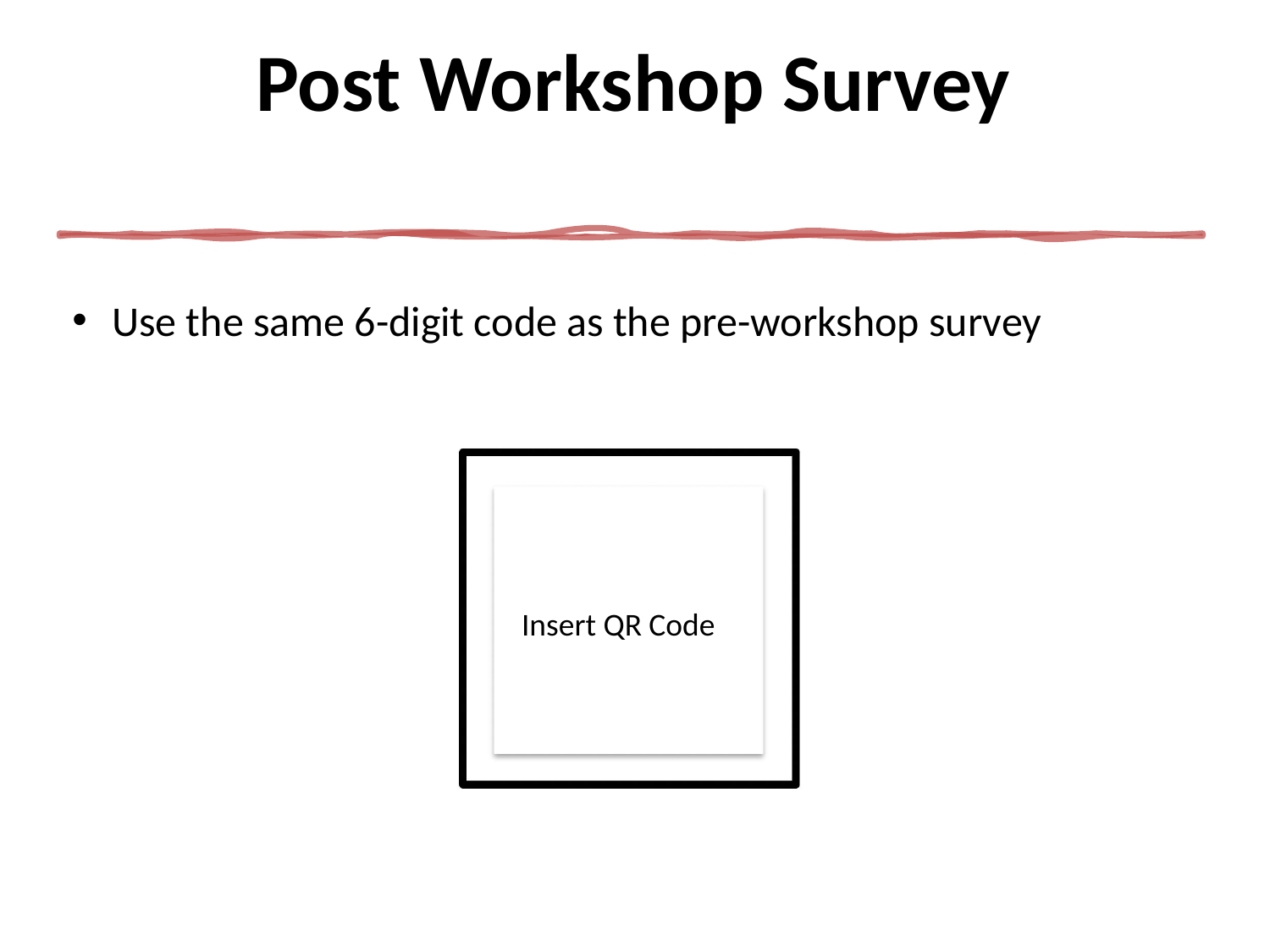

# Post Workshop Survey
Use the same 6-digit code as the pre-workshop survey
Insert QR Code

## Slide 33
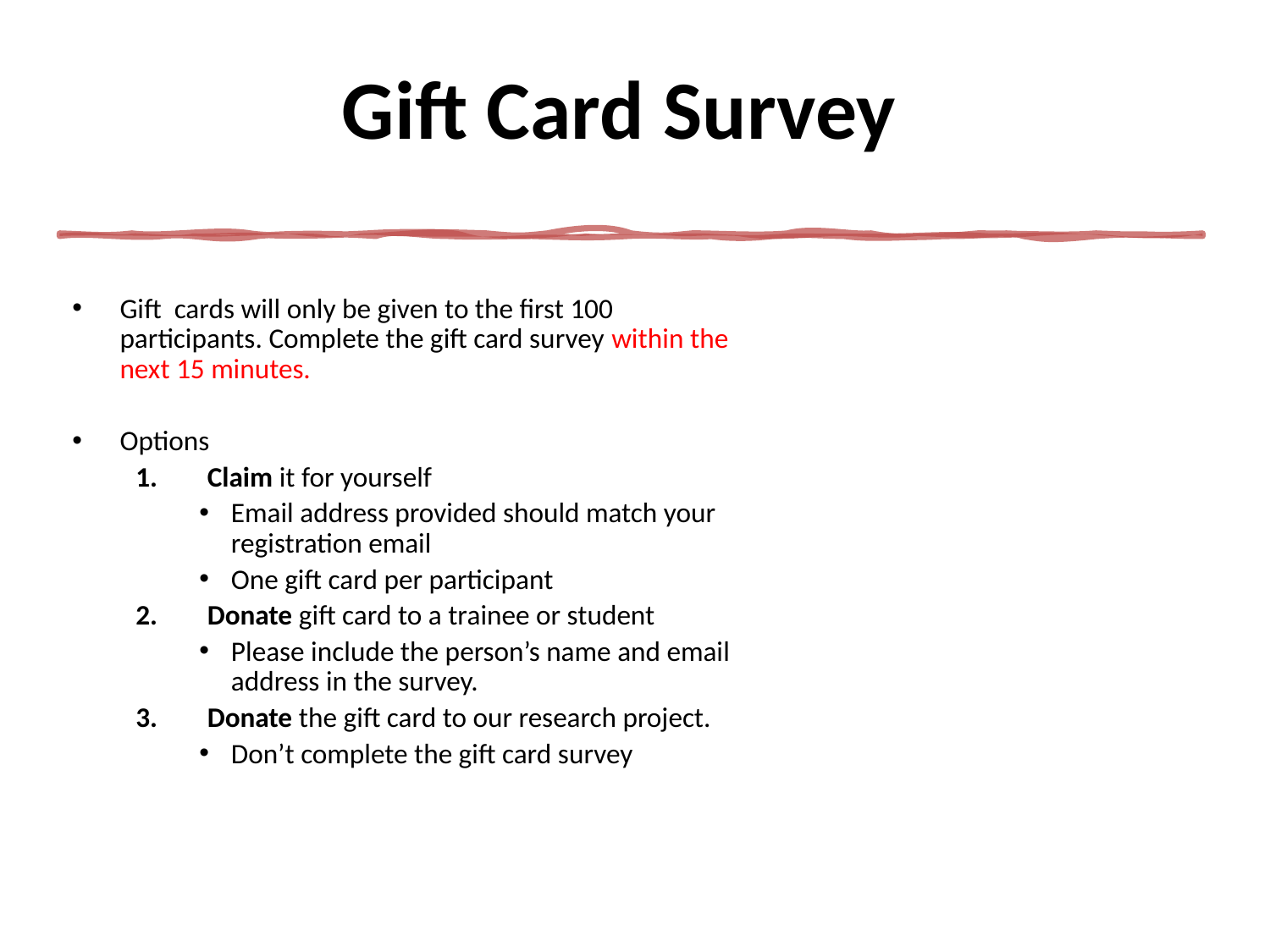

# Gift Card Survey
Gift cards will only be given to the first 100 participants. Complete the gift card survey within the next 15 minutes.
Options
Claim it for yourself
Email address provided should match your registration email
One gift card per participant
Donate gift card to a trainee or student
Please include the person’s name and email address in the survey.
Donate the gift card to our research project.
Don’t complete the gift card survey

## Slide 34
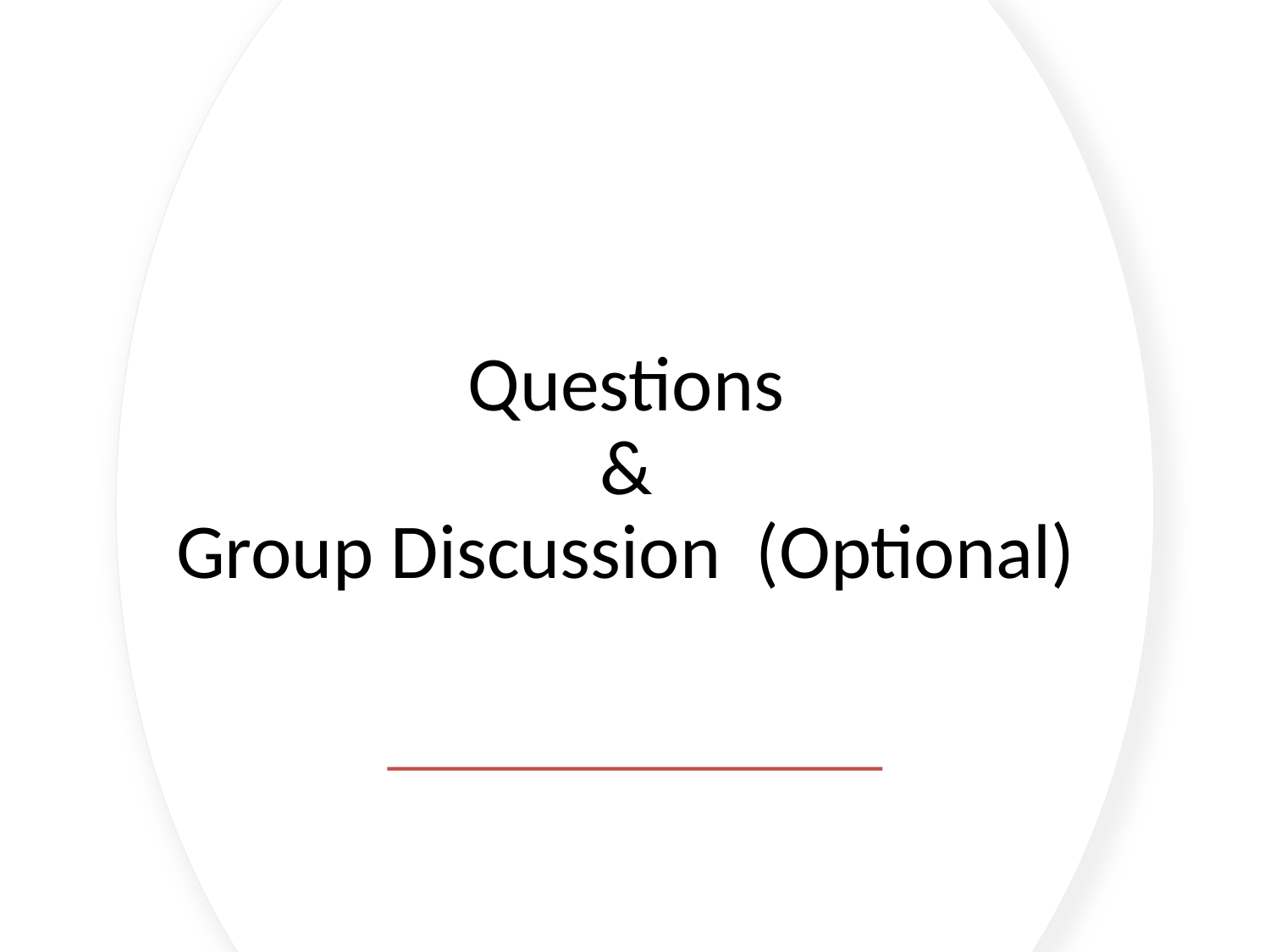

# Questions & Group Discussion (Optional)
